# The Lysine Acetyltransferase Activator Brpf1 Governs Dentate Gyrus Development through Neural Stem Cells and Progenitors

**DOI:** 10.1371/journal.pgen.1005034

**Published:** 2015-03-10

**Authors:** Linya You, Kezhi Yan, Jinfeng Zhou, Hong Zhao, Nicholas R. Bertos, Morag Park, Edwin Wang, Xiang-Jiao Yang

**Affiliations:** 1 The Rosalind & Morris Goodman Cancer Research Center, McGill University, Montreal, Quebec, Canada; 2 Department of Medicine, McGill University, Montreal, Quebec, Canada; 3 Department of Biochemistry, McGill University, Montreal, Quebec, Canada; 4 National Research Council Canada, Montreal, Quebec, Canada; 5 McGill University Health Center, Montreal, Quebec, Canada; Massachusetts General Hospital, Howard Hughes Medical Institute, UNITED STATES

## Abstract

Lysine acetylation has recently emerged as an important post-translational modification in diverse organisms, but relatively little is known about its roles in mammalian development and stem cells. Bromodomain- and PHD finger-containing protein 1 (BRPF1) is a multidomain histone binder and a master activator of three lysine acetyltransferases, MOZ, MORF and HBO1, which are also known as KAT6A, KAT6B and KAT7, respectively. While the *MOZ* and *MORF* genes are rearranged in leukemia, the *MORF* gene is also mutated in prostate and other cancers and in four genetic disorders with intellectual disability. Here we show that forebrain-specific inactivation of the mouse *Brpf1* gene causes hypoplasia in the dentate gyrus, including underdevelopment of the suprapyramidal blade and complete loss of the infrapyramidal blade. We trace the developmental origin to compromised Sox2^+^ neural stem cells and Tbr2^+^ intermediate neuronal progenitors. We further demonstrate that Brpf1 loss deregulates neuronal migration, cell cycle progression and transcriptional control, thereby causing abnormal morphogenesis of the hippocampus. These results link histone binding and acetylation control to hippocampus development and identify an important epigenetic regulator for patterning the dentate gyrus, a brain structure critical for learning, memory and adult neurogenesis.

## Author Summary

Lysine acetylation refers to addition of the acetyl group to lysine residues after protein synthesis. Little is known about how this modification plays a role in the brain and neural stem cells. It is catalyzed by a group of enzymes known as lysine acetyltransferases. A novel epigenetic regulator called BRPF1 acts as a master activator of three different lysine acetyltransferases and also contains multiple structural domains for histone binding. In this study, we show that forebrain-specific inactivation of the mouse *Brpf1* gene causes abnormal development of the dentate gyrus, a key component of the hippocampus. We trace the developmental origin to compromised neural stem cells and progenitors, and demonstrate that Brpf1 loss deregulates neuronal migration and cell cycle progression during development of the dentate gyrus. This is the first report on an epigenetic regulator whose loss has such a profound impact on the hippocampus, especially the dentate gyrus, a brain structure critical for learning, memory and adult neurogenesis.

## Introduction

Lysine acetylation involves covalent addition of an acetyl moiety to the ε-amino group of a lysine residue and is important for modification of both prokaryotic and eukaryotic proteins [1–3]. Proteomic analyses have detected this modification in thousands of mammalian proteins with important roles not only in chromatin-templated nuclear processes but also various cytoplasmic pathways [4–7]. In addition, it is abundant in bacteria [8,9]. Although some of the modification events in bacteria are dependent on acetyl-phosphate [10], this modification is exclusively enzymatic in eukaryotes. In humans, at least 15 known lysine acetyltransferases (KATs) catalyze the forward reaction [2,11–13]. These enzymes are divided into three families, one of which is the MYST family, composed of TIP60 (HIV Tat-interacting protein of 60 kDa), MOZ (monocytic leukemia zinc finger protein), MORF (MOZ-related factor), HBO1 (HAT bound to ORC1) and hMOF (homolog of *Drosophila* males absent on the first), which are also known as KAT5, KAT6A/MYST3, KAT6B/MYST4, KAT7/MYST2 and KAT8/MYST1, respectively [14–16]. Although mainly referred to as histone acetyltransferases, members of this family also acetylate non-histone substrates, including the tumor suppressor p53 [17–19] and the DNA-damage response regulator DBC1 (deleted in breast cancer 1) [20]. TIP60 and hMOF also carry out autoacetylation essential for their activation [21–25]. Furthermore, four recent studies have revealed that tyrosine phosphorylation of TIP60 links chromatin sensing to ATM signaling and that both TIP60 and hMOF regulate autophagy [26–29]. Thus, this family of acetyltransferases is important in diverse cellular programs.

Molecular and cell-based studies have firmly established that three members of this family, MOZ, MORF and HBO1, form tetrameric complexes with BRPF1 (bromodomain- and PHD finger-containing protein 1), along with two other subunits [30–32]. Within the complexes, BRPF1 functions as a scaffold to bridge subunit interaction, stimulate acetyltransferase activity and restrict substrate specificity [30–32]. Moreover, BRPF1 possesses two PHD fingers for binding to unmodified histone H3 [32], one bromodomain for acetyllysine-recognition [33] and a PWWP domain for specific interaction with methylated histone H3 [34,35]. Thus, BRPF1 is a unique multivalent histone binder able to activate different acetyltransferases.

BRPF1 is highly conserved from *Drosophila* to humans [reviewed in 16]. In *C*. *elegans*, a distantly related protein, Lin-49, regulates neuron asymmetry, hindgut development and fecundity [36–38]. In vertebrates, BRPF1 has two paralogs, BRPF2 and BRPF3 [30,31]. Inactivation of zebrafish *Brpf1* alters pharyngeal segmental identity [39], and disruption of medaka fish *Brpf1* affects craniofacial and caudal skeletons [40], indicating that fish Brpf1 regulates skeletal development. These studies suggest that mammalian BRPF1 may also play an important role in development. Of relevance, loss of mouse Brpf2 leads to embryonic lethality, with growth retardation, neural tube defects, abnormal eye development and faulty erythropoiesis [41], supporting that BRPF1, BRPF2 and BRPF3 have non-redundant functions *in vivo*.

As key partners of BRPF1, MOZ and MORF are important in different types of normal and pathological stem cells. Mouse Moz plays a key role in self-renewal and maintenance of hematopoietic stem cells [42,43]. Consistent with this, human *MOZ* and *MORF* are rearranged in leukemia and other hematological malignancies [reviewed in 14,16]. One of the resulting fusion proteins is crucial for self-renewal of leukemic stem cells [44,45]. In addition, the *MORF* gene is frequently altered in castration-resistant prostate cancer [46] and its mutations have been detected in breast cancer [47], although it remains unclear whether related cancer stem cells are affected. The gene is also mutated in four developmental disorders, Noonan syndrome-like disorder [48], Ohdo syndrome [49] and genitopatellar syndrome [50,51] and blepharophimosis-ptosis-epicanthus inversus syndrome [52]. One common characteristic of these disorders is intellectual disability. Related to this, mice with residual *MORF* expression display neocortical defects [53] and abnormal neural stem cells [54], raising an interesting question whether BRPF1 plays a role in the brain.

The interaction of BRPF1 with MOZ and MORF, as indicated by molecular and cell-based studies [30–32], suggests the exciting possibility that BRPF1 may regulate mammalian development. Related to this, we have recently found that it is essential for mouse embryo survival [55]. Our expression survey has also identified high-level expression of Brpf1 in the brain [55]. Here we examine this further and demonstrate that forebrain-specific inactivation of the mouse *Brpf1* gene leads to dentate gyrus hypoplasia, reduces expression of key genes involved and deregulates neural stem cells and progenitors. Upon Brpf1 loss, Sox2^+^ neural stem cells and Tbr2^+^ intermediate neuronal progenitors fail to settle at the subgranular zone of the dentate gyrus, one of the two major sites known to harbor adult neural stem cells. This is the first epigenetic regulator to be identified with such an important role in the dentate gyrus.

## Results

### Dynamic expression of Brpf1 during mouse forebrain development

**Fig 1:**
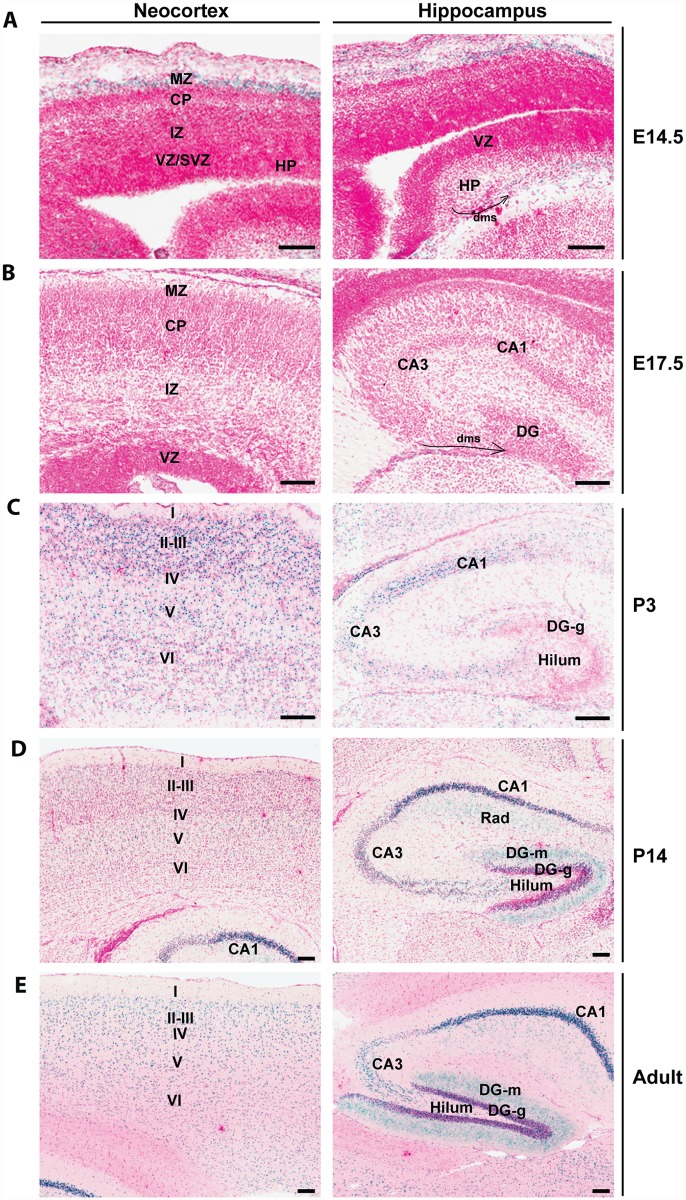
*Brpf1* expression during forebrain development. (A) At E14.5, β-galactosidase activity (labeled blue, with nuclei stained red) was detected in the marginal zone (MZ) of the cortical hem, but not in the developing neocortex. In the hippocampus, the signal was detected sparsely in the dentate migration stream (dms, marked by a curved arrow). (B) At E17.5, the expression is very weak in the neocortex and hippocampus. Note the sparse signals detected in the dentate migration stream (dms) marked by a curved arrow. (C-E) After birth, β-galactosidase activity appeared in the neocortex and hippocampus. From P3 onwards, β-galactosidase activity was detected in all the six layers of neocortex and in the cornum amonni (CA) field of the hippocampus. The activity in the dentate gyrus was moderate at P3 but became much stronger at P14 and adult. All images were taken from frozen sections prepared from *Brpf1* ^*l/+*^ embryos (A-B) or postnatal brains (C-E); the corresponding wild-type sections showed no β-galactosidase staining [55]. Structures of the developing and adult brains were annotated according to published atlases [87–91]. CA1, cornum amonni 1; CA3, cornum amonni 3; CP, cortical plate; DG, dentate gyrus; DG-g, granular cell layer of dentate gyrus; DG-m, molecular layer of dentate gyrus; HP, hippocampus; I-IV, cortical layers I-VI; IZ, intermediate zone; Rad, radial layer of the hippocampus; SVZ, subventricular zone; VZ, ventricular zone. Scale bars, 100 μm.

By using a mouse strain containing a *LacZ* knockin cassette inserted at the *Brpf1* locus (S1A Fig), we have recently detected high β-galactosidase activities in the neocortex and hippocampus [55]. To gain further insights, we examined frozen sections prepared from *Brpf1* ^*l/+*^ pre- and post-natal brains more carefully than previously reported [55]. As shown in Fig. 1A (left), strong expression was detected at the marginal zone of the developing neocortex at embryonic day (E) 14.5. At E17.5, very weak expression was present in different regions of the neocortex (Fig. 1B, left). At postnatal day (P) 3, P14 and the adult stage, much stronger expression was found in all six layers of the neocortex, with some enrichment in layers II-III (Fig. 1C-E, left). Similarly, we examined the expression in the hippocampus. At E14.5 and E17.5, no expression was detectable in the hippocampal primordium (Fig. 1A-B, right). Interestingly, at both stages, sparse signals were present in the dentate migration stream (Fig. 1A-B, right). At P3, relatively strong expression appeared in the cornum amonni (CA) regions (Fig. 1C, right), indicating that Brpf1 expression increases dramatically from E17.5 to P3. At P3, only sparse signals were detected in the developing dentate gyrus (Fig. 1C, right). At P14 and the adult stage, strong expression was detected in the dentate gyrus (Fig. 1D-E, right). These results support a potential role of Brpf1 in neocortex and hippocampus development.

### Generation of forebrain-specific *Brpf1* knockouts

To determine the function of Brpf1 in the forebrain, we crossed heterozygous *Brpf1* ^*f/+*^ mice (S1A Fig) with the Emx1-Cre strain, which expresses the Cre recombinase from the Emx1 locus and allows LoxP excision in the neocortex and hippocampus [56]. The resulting *Brpf1* ^*f/+*^ *;Emx1-Cre* mice appeared normal and intercrosses between them yielded *Brpf1* ^*f/f*^;*Emx1-Cre* (or bKO, short for forebrain conditional knockout) animals. Genomic PCR (S1B Fig) and RT-PCR (S1C Fig) confirmed specific excision in the forebrain but not cerebellum. The specific knockout had minimal effects on the expression of Brpf2, Brpf3, Moz and Morf (S1D Fig). Most of the bKO pups died prior to weaning at P21 [57]. Systematic histological analysis of the mutant brain identified three defective areas: the neocortex, hippocampus and corpus callosum, whereas other brain regions such as the cerebellum were normal [57]. Among these defects, the one in the hippocampus is the most striking and thus investigated here.

### Brpf1 loss leads to partial agenesis of the hippocampus

**Fig 2:**
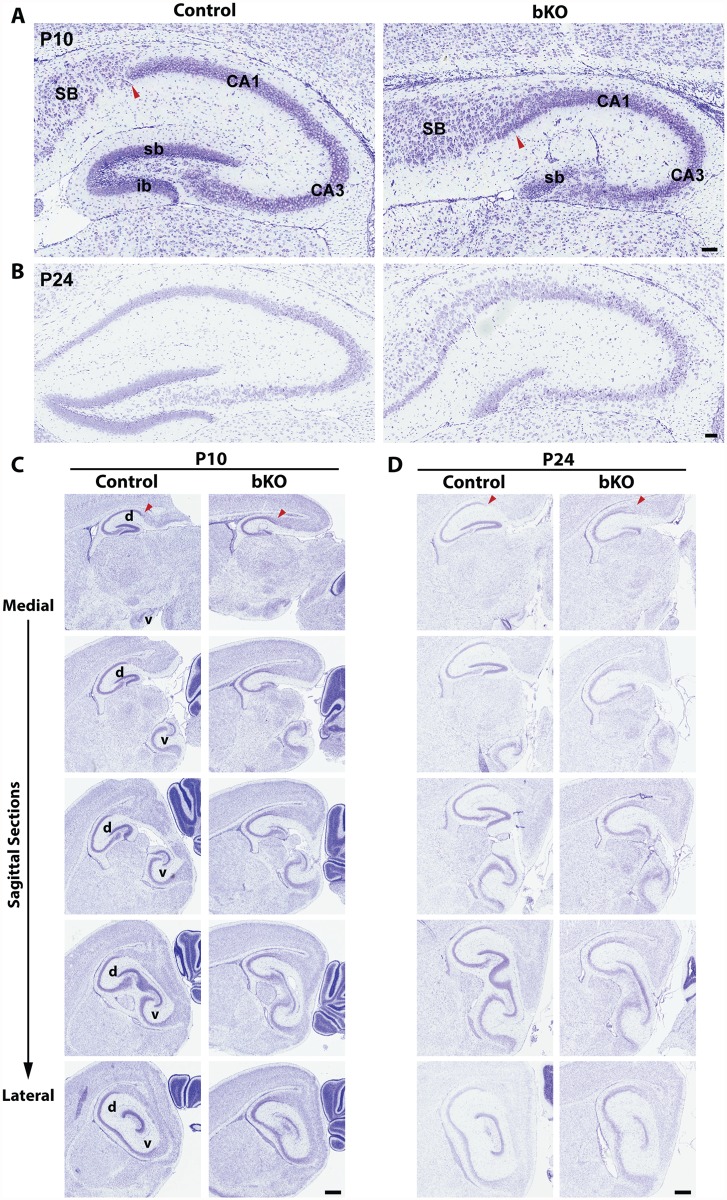
Forebrain-specific Brpf1 loss causes hypoplasia of the dentate gyrus. (A-B) Nissl staining was performed on coronal brain sections from P10 and P24 mice. Loss of *Brpf1* resulted in underdevelopment of the suprapyramidal blade (sb) and disappearance of the infrapyramidal blade (ib) in the dentate gyrus. The border between cornu ammonis 1 (CA1) and the subiculum (SB) was clearly defined in the wild-type sections but became obscure in the mutant, as indicated by red arrowheads in (A). At P10 and P24, the pyramidal layers of CA1 and CA3 in the mutant sections were not as tightly packed as in the wild-type (A-B). (C-D) Nissl staining of serial brain sections. Five medial-to-lateral sagittal sections were prepared from P10 (C) or P24 (D) wild-type and mutant brains and Nissl-stained to analyze the morphology of the hippocampus at different planes (A). The border between CA1 and the subiculum, marked by red arrowheads, was clearly defined in the wild-type but not mutant sections. v, ventral hippocampus; d, dorsal hippocampus. Scale bars, 100 μm for (A-B) and 0.5 mm for (C-D).

**Fig 3:**
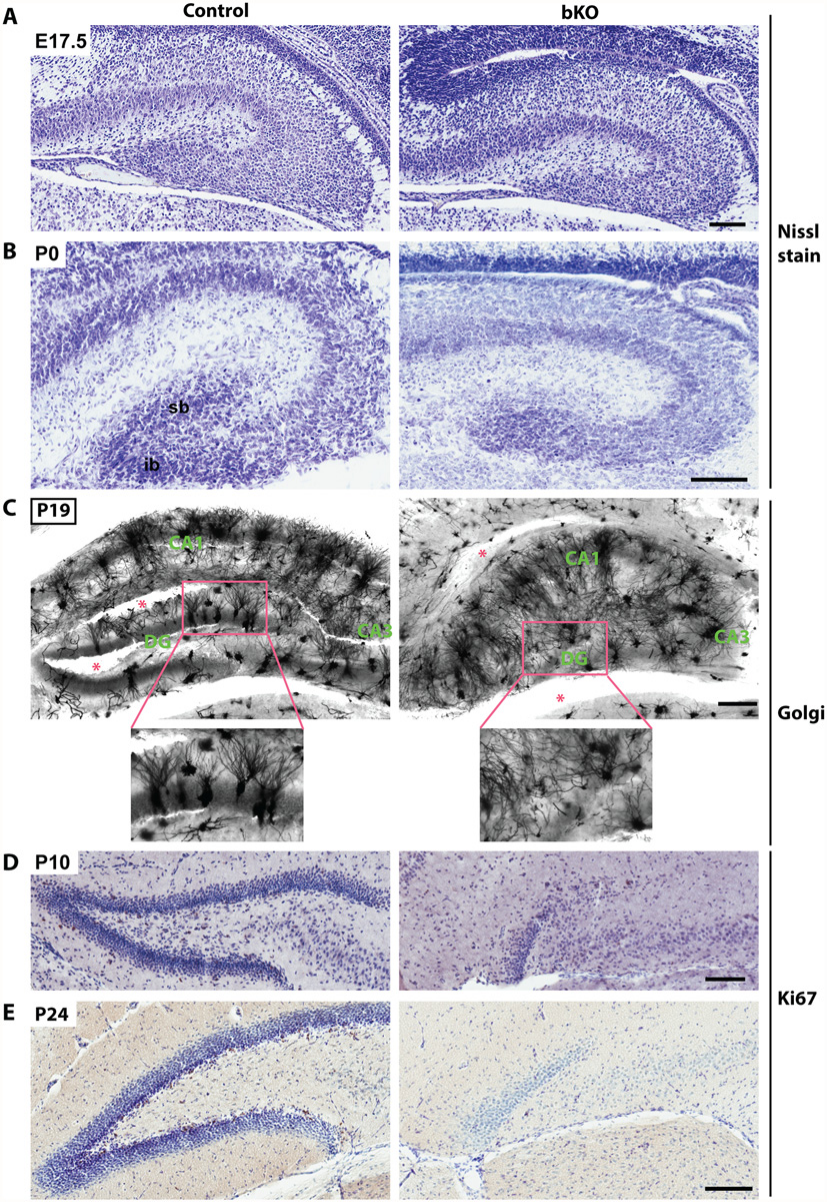
Brpf1 loss impairs dentate gyrus development, dendritic tree formation and neuronal proliferation. (A-B) Nissl staining of coronal brain sections from E17.5 and P0 mice. At P0, loss of *Brpf1* resulted in underdevelopment of the suprapyramidal blade (sb) and disappearance of the infrapyramidal blade (ib) in the developing dentate gyrus. (C) Golgi-Cox staining of coronal brain sections at P19. Representative images of hippocampal regions from the wild-type and bKO brain sections show that the bKO hippocampus possessed disorganized neurons, with less robust dendritic trees. There were also fewer neurons in the mutant dentate gyrus. Red asterisks denote areas accidentally torn during staining. The boxed regions in the top panels are shown in the lower panels at higher magnification. (D-E) Ki67 immunohistochemistry showing that cell proliferation dramatically decreased in the subgranular zone of the bKO dentate gyrus at P10 and P24. The subgranular zones of the control P10 and P24 sections shown here contain 20 and 34 Ki67^+^ cells, respectively, whereas the corresponding regions of the mutant sections possess either one or no Ki67^+^ cells. Scale bars, 100 μm for (A-B & D-E) and 200 μm for (C).

Nissl staining of brain sections revealed that when compared to the control, the suprapyramidal blade of the dorsal hippocampus in the P10 mutant brain was shorter, with one end remaining attached to the ventricular zone, whereas the infrapyramidal blade was completely missing (Fig. 2A, right). The nuclear layers of CA1 and CA3 appeared more diffusely packed than those in the control (Fig. 2A, right). In the mutant, the junction of CA1 with the subiculum was not as clear-cut as that in the control and the subiculum itself was expanded. Similar changes were found in the mutant brain at P24 (Fig. 2B). More importantly, these defects also appeared in serial sagittal sections and similar abnormalities were found in the ventral hippocampus (Fig. 2C-D), indicating that the entire hippocampal formation is affected. The mouse dentate gyrus develops from the cortical hem around mid-gestation and involves dynamic neuron migration and differentiation, both of which continue in the first two weeks after birth [58,59]. To determine the developmental point when the defects start to occur, we applied Nissl staining to brain sections from E17.5 fetuses and P0 neonates. As shown in Fig. 3A-B, the developing dentate gyrus was underdeveloped at both time points, indicating that the defects originate from prenatal development.

To characterize the defects further, we performed Golgi-Cox staining to assess formation of axons and dendrites. This staining revealed abnormal axon and dendritic trees in the hippocampus (Fig. 3C). Interestingly, the disorganization was not just limited to the dentate gyrus but also occurred in the CA regions, indicating that Brpf1 is required for proper development of the entire hippocampus. Another method to examine the axons of the dentate granule cells, the so-called mossy fibers, is the Timm’s stain. The first mossy fibers invade CA3 at around P0, and their number gradually increases during postnatal development [60]. We examined mossy fiber development at P8 by Timm’s stain and found that the fiber projection was defective from the very beginning of its development. The suprapyramidal bundles (spb) and the dentate hilum of mossy fibers were virtually missing in the mutant (S2 Fig).

To shed light on the underlying mechanisms for the defects, we examined the sections by immunohistochemical staining with an anti-Ki67 antibody. As shown in Fig. 3D-E, Ki67^+^ neuronal precursors were enriched in the subgranular zone of the wild-type dentate gyrus at both P10 and P24 (left), but such precursors were missing in the mutant (right), indicating a lack of proliferation in the subgranular zone. These results indicate that Brpf1 is essential for hippocampus development and regulates neuronal proliferation in the dentate gyrus.

### Brpf1 loss deregulates neural stem cells and progenitors

To gain mechanistic insights into the observed defects in the hippocampus, we asked whether there are other mutant mice displaying similar phenotypes in the dentate gyrus. Literature search revealed that loss of several transcription factors cause similar hypoplasia in the dentate gyrus, including Sox2 [61], Tlx (tailless) [62], Tbr2 (T-box brain protein 2, also known as eomesodermin) [63], NeuroD1 [64], Emx2 (empty spiracles homeobox 2) [65], neurogenin 2 [66] and FoxG1 (also known as BF1, for brain factor 1) [67]. Among these, Sox2 and Tlx are two well-known neural stem cell markers [68,69], whereas Tbr2 is important for intermediate neuronal progenitors [70]. The unexpected finding that upon loss, Brpf1 shares phenotypes with these three transcription factors in dentate gyrus development suggests a potential link to neural stem cells and progenitors.

**Fig 4:**
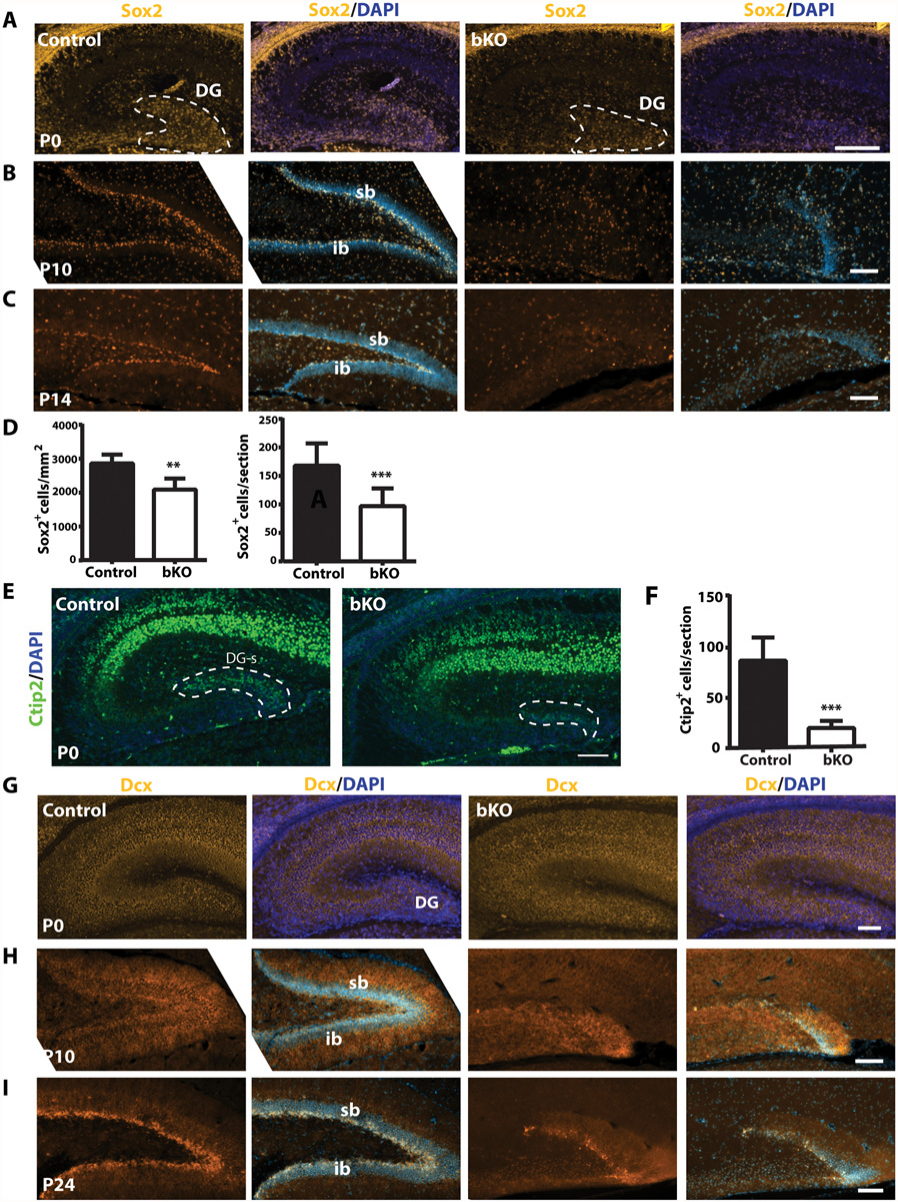
Brpf1 loss compromises neural stem cells and neuronal precursors. (A-C) Immunofluorescence microscopy to detect Sox2^+^ neural stem cells (NSCs) on peri- or postnatal brain sections. At P0, Sox2^+^ NSCs were enriched in the wild-type dentate gyrus (DG) and this population was smaller in the mutant, as quantified in (D). At P10 and P14, Sox2^+^ NSCs settled in the control subgranular zone (SGZ), while in the mutant, the granule cell layers were hypoplastic and the SGZ harbored few Sox2^+^ NSCs. (D) Quantification of Sox2^+^ cells in the control and mutant dentate gyri at P0. There were significantly fewer Sox2^+^ NSCs within the mutant dentate gyrus per section (right). The number of Sox2^+^ cells per mm^2^ within the dentate gyrus also significantly decreased (left). The quantification was based on three pairs of neonates and at least three matched sections per brain. **p<0.01; ***p<0.001. (E) In the hippocampus, Ctip2 expression was restricted to the CA regions and the suprapyramidal blade of the developing dentate gyrus (DG-s) at P0. (F) Quantification of Ctip2^+^ cells in the wild-type and mutant suprapyramidal blades, outlined in (E), was based on three pairs of neonates and at least three matched sections per brain. ***p<0.001. (G-I) Dcx expression in control and bKO brain sections at three developmental stages. In the mutant dentate gyrus, there were fewer Dcx^+^ neuronal precursors apparently at P10 (H) and P24 (I). Scale bars: (A-C), 100 μm; (E), 400 μm, (G-I), 100 μm.

The subgranular zone of the dentate gyrus is one of two major sites harboring adult neural stem cells [68]. As noted above, Sox2 is a neural stem cell marker and its loss leads to dentate gyrus hypoplasia [61]. In addition, Ki67^+^ neuronal precursors disappeared in the subgranular zone of the mutant dentate gyrus (Fig. 3D-E). These observations suggest that Brpf1 loss may deregulate neural stem cells. To investigate this possibility, we performed immunostaining of brain sections with an antibody against Sox2. At P0, Sox2^+^ neural stem cells were enriched in the wild-type dentate gyrus (Fig. 4A, left two panels). This population was smaller in the mutant dentate gyrus (Fig. 4A, right two panels & Fig. 4D). In support of this, when compared to the wild-type dentate gyrus, the mutant contained a much smaller population of neurons expressing Ctip2 (Fig. 4E-F), a transcription factor important for dentate gyrus development [71]. As development progressed to P10 and P14, wild-type Sox2^+^ neural stem cells became enriched in the subgranular zone (Fig. 4B-C, left two panels). By contrast, no such enrichment was present in the mutant (Fig. 4B-C, right two panels), suggesting the requirement of Brpf1 for development of Sox2^+^ neural stem cells in the dentate gyrus.

We also analyzed doublecortin (Dcx)-expressing neuroblasts. At P0, Dcx^+^ cells were enriched in both wild-type and mutant dentate gyri (Fig. 4G), and the distribution was rather uniform in the developing dentate gyrus. Different from Sox2^+^ neural stem cells, Dcx^+^ immature neurons were also present in the pryramidal layers of the CA1 and CA3 regions (Fig. 4G). As the development progressed, Dcx^+^ immature neurons became enriched in the subgranular zone of the wild-type dentate gyrus at P10 and P24 (Fig. 4H-I, left panels). A similar trend of enrichment was observed in the mutant, but the cell number was greatly reduced (Fig. 4H-I, right panels), supporting that Brpf1 loss comprises Dcx^+^ neuroblasts.

**Fig 5:**
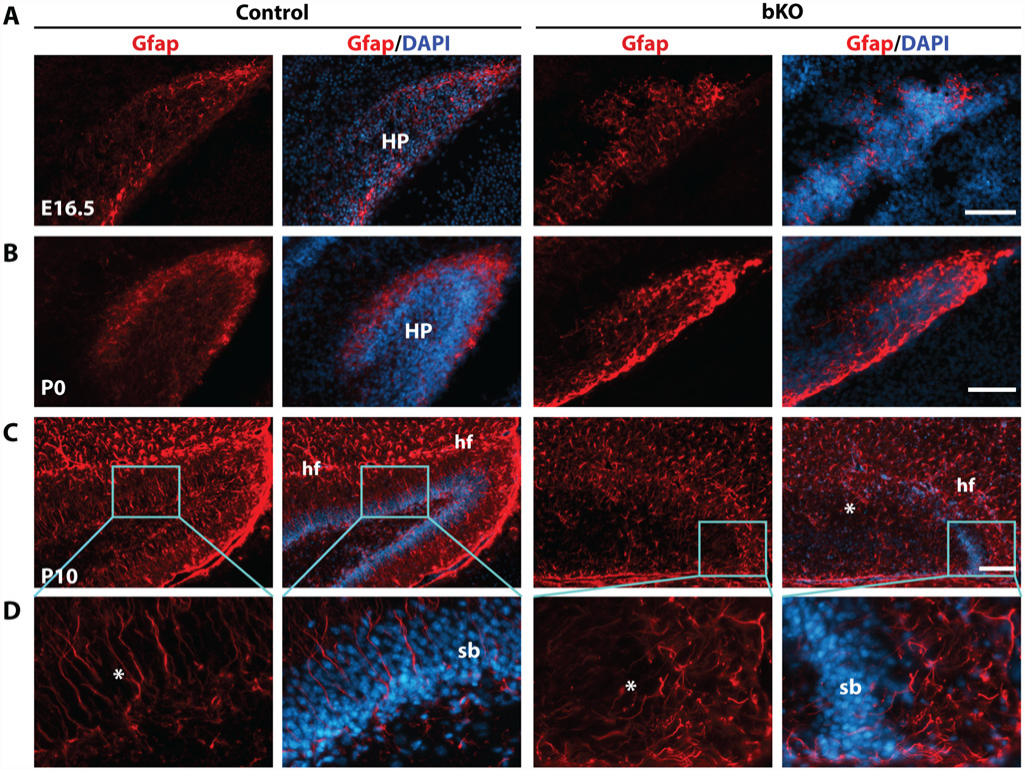
Deregulated Gfap expression in the mutant hippocampus. (A-C) Immunofluorescence microscopy to detect Gfap expression on brain sections at E16.5, P0 and P10. Distribution of Gfap^+^ radial glial cells in the mutant dentate gyri was only slightly disturbed at E16.5 and moderately at P0 (A-B), but it became completely disorganized at P10 (C). (D) High-magnification images of the regions boxed in (C). Compared to the wild-type dentate gyrus, there were few Gfap^+^ radial glial cells in the mutant (see the areas marked by asterisks). In addition, the hippocampus fissure (hf) was ill-formed in the mutant. HP, hippocampus; sb, suprapyramidal blade; scale bars, 100 μm.

Intermediate neuronal progenitors are essential for dentate gyrus development [72,73]. Tbr2 is a marker of these progenitors. The similar phenotype of *Tbr2* inactivation in the dentate gyrus [63] suggests that these progenitors may also be affected. Intermediate neuronal progenitors are derived from radial glial cells [70], so we first analyzed the expression of Gfap (glial fibrillary acidic protein), a well-known marker of radial glial cells [73]. At E16.5, distribution of Gfap^+^ glial cells was slightly altered in the mutant dentate gyrus (Fig. 5A). At P0, such cells were enriched at the outer rim of the wild-type dentate gyrus (Fig. 5B, left two panels), but this distribution became disorganized in the mutant (right two panels). At P10, Gfap^+^ radial glial cells and astrocytes were nicely organized along the granular cell layer of the dentate gyrus and the hippocampal fissure, respectively (Fig. 5C, left two panels), but this pattern became virtually missing in the mutant (right two panels). In particular, there were fewer Gfap^+^ radial glial cells in the granular cell layer of the mutant dentate gyrus and these cells were disoriented (Fig. 5D).

**Fig 6:**
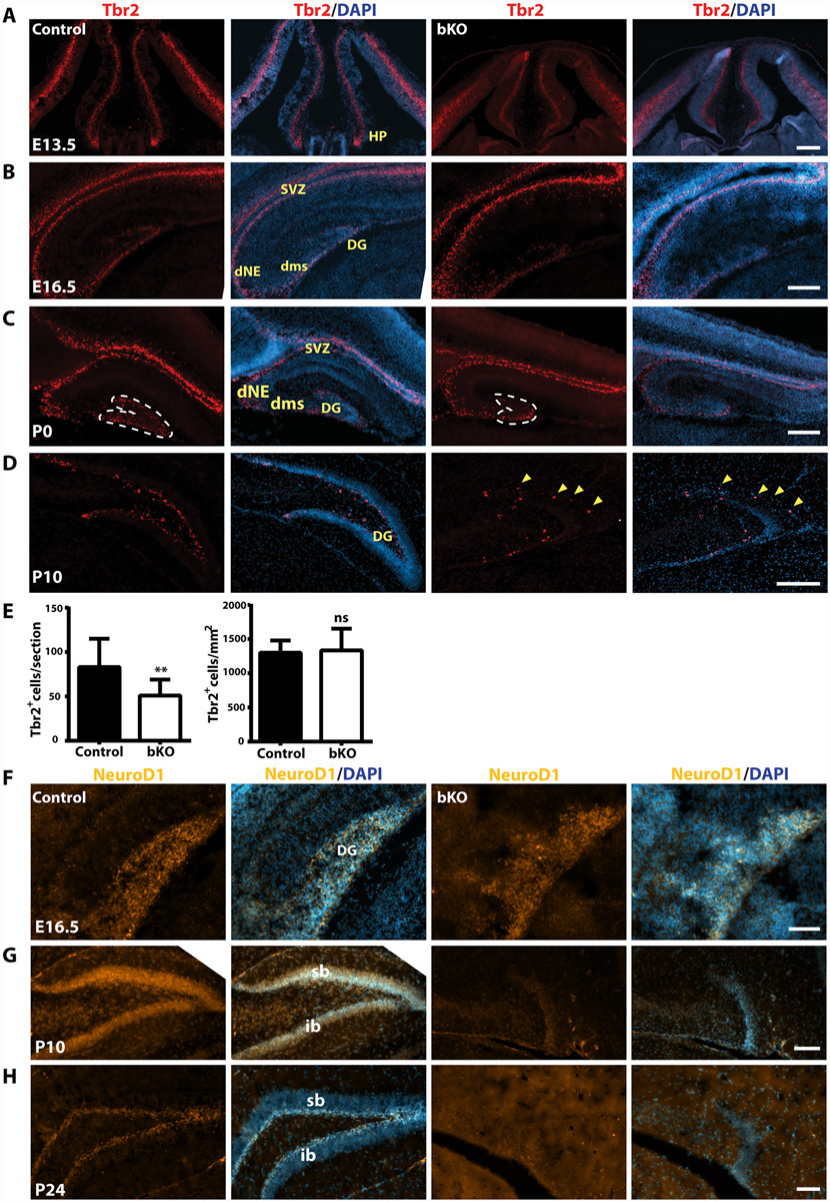
Defective Tbr2^+^ and NeuroD1^+^ neuronal precursors in the mutant hippocampus. (A-D) Immunostaining to detect Tbr2^+^ intermediate neuronal progenitors on sections from E13.5, E16.5, P0 and P10 brains. At E13.5 (A), Tbr2 was similarly expressed in the control and mutant neuroepithelium and hippocampus primordium (HP). At E16.5 (B), Tbr2 was similarly expressed in the control and mutant subventricular zones (SVZ) and hippocampi. At P0 and P10, there were fewer Tbr2^+^ progenitors in the mutant dentate gyrus (C-D). The yellow arrowheads in the right two panels mark four progenitors that failed to settle in the subgranular zone, but stayed at the outer rim of the molecular cell layer. (E) Quantification of Tbr2^+^ progenitors in the developing dentate gyrus at P0, outlined with clear dash lines in (C). While there was no significant difference in the cell density (right), the Tbr2^+^ progenitor number per section decreased significantly in the mutant (left). The quantification was based on three pairs of control and mutant brains, with at least three matched sections per brain. **p<0.01; ns, not statistically significant. (F-H) Immunofluorescence microscopy to detect NeuroD1^+^ neuroblasts on E16.5, P10 and P24 brain sections. At E16.5 (F), NeuroD1 expression was relatively normal in the mutant hippocampus (HP), but it virtually disappeared in the mutant dentate gyrus at P10 (G) and P24 (H). DG, dentate gyrus; dNE, dentate neuroepithelium; dms, dentate migration stream; HP, hippocampus; sb, suprapyramidal blade; ib, infrapyramidal blade; SVZ, subventricular zone; scale bars, 100 μm.

We next analyzed intermediate neuronal progenitors with an anti-Tbr2 antibody. At E13.5, Tbr2 expression in the hippocampal primordium was rather similar between the wild-type and mutant (Fig. 6A). By E16.5, Tbr2^+^ progenitors were present in the wild-type dentate neuroepithelium and migrated along the dentate migratory stream to the forming dentate gyrus (Fig. 6B, left two panels). Such migration was also found in the mutant, but the population was smaller in the forming dentate gyrus (Fig. 6B, right two panels). At P0, a similar difference was found between the wild-type and mutant dentate gyri (Fig. 6C). Quantification confirmed this (Fig. 6E). As the development progressed, Tbr2^+^ progenitors translocated to the subgranular zone and the hilum of the wild-type dentate gyrus (Fig. 6D, left two panels). In stark contrast, no such translocation was found in the mutant (right two panels), with some of Tbr2^+^ progenitors stayed at the molecular cell layer (Fig. 6D, right two panels; marked with yellow arrowheads). These results indicate that the abnormalities start at a fetal stage and *Brpf1* inactivation impairs the migration of Tbr2^+^ progenitors.

We performed additional analyses with antibodies against three neurogenic transcription factors, NeuroD1, neurogenin 2 and FoxG1. At E16.5, NeuroD1^+^ neuroblasts were enriched in both wild-type and mutant developing dentate gyri, rather uniformly distributed across the granular layer and the hilum (Fig. 6F). As the development progressed to P10, NeuroD1^+^ neuroblasts became enriched in the granular cell layer in the wild-type dentate gyrus (Fig. 6G, left panels). By contrast, few such cells were present in the mutant dentate gyrus (right panels). At P24, NeuroD1^+^ neuroblasts were restricted to the wild-type subgranular zone (Fig. 6H, left panels), but almost none was observed in the mutant (right panels). These results indicate that Brpf1 is required for proper development and migration of NeuroD1^+^ neuroblasts.

Neurogenin 2 is essential for dentate gyrus formation [66]. At P0, distribution of neurogenin 2^+^ progenitors was ubiquitious in the wild-type and mutant hippocampi (S3A Fig). At P24, all granular neurons in the dentate gyrus and the pyramidal neurons in the CA1 and CA3 regions expressed neurogenin 2 (S3B Fig, left two panels). This expression pattern was also observed in the mutant, but the cells were not as tightly packed in the pyramidal layers of the CA regions and the suprapyramidal blade of the dentate gyrus (right two panels). As expected, the infrapyramidal blade was missing. For immunostaining to detect the brain-specific transcription factor FoxG1, we performed immunostaining with antibodies against FoxG1 and Tuj1, a neuron-specific β-tubulin. This analysis revealed no obvious defects at E13.5 (S4A Fig). At P0, the hippocampal region became slightly disorganized (S4B Fig). At P24, for the wild-type hippocampus, FoxG1 was expressed in pyramidal neurons of the CA1 region and the granular layer of the dentate gyrus, but not in pyramidal neurons of the CA3 region (S4C Fig, left two panels). For the mutant hippocampus, FoxG1 was also expressed in these two regions (S4C Fig, right two panels), but the neurons were much less tightly packed than those in the wild-type hippocampus (S4C Fig). Moreover, unlike the wild-type, FoxG1 expression was also detected in pyramidal neurons of the CA3 region (S4C Fig). As expected, the infrapyramidal blade was absent in the mutant hippocampus (S4C Fig, right two panels). These results support that *Brpf1* inactivation alters neurogenesis in the hippocampus.

It is interesting to note that the impact on Sox2, Tbr2 and NeuroD1 (Figs. 4A-C & 6) is quite different from that on neurogenin 2 and FoxG1 (S3–S4 Figs). With the former three, the positively expressing neural stem cells or neuronal precursors displayed active migration in the dentate gyrus and settled at the subgranular zone (Figs. 4A-C & 6). By contrast, neurogenin 2-expressing progenitors or FoxG1-expressing neurons were rather uniformly distributed in the granular layer (S3–S4 Figs). These two groups of transcription factors appear to regulate different stages of dentate gyrus development. Thus, Brpf1 may regulate different stages of hippocampus development.

### Brpf1 loss impairs neuronal migration and cell cycle progression

**Fig 7:**
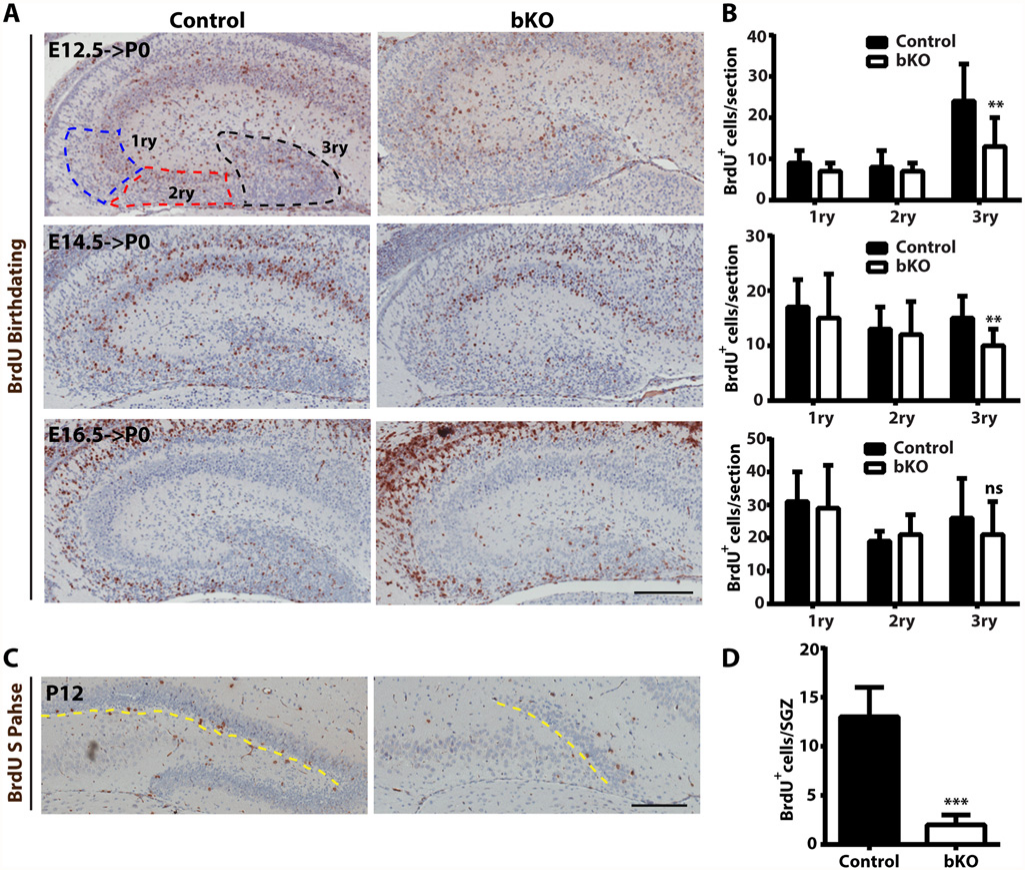
Analysis of neuronal migration in the hippocampus by BrdU labeling. (A-B) After injection with BrdU at E12.5, E14.5 and E16.5, pregnant mice were sacrificed at P0 for immunohistochemical analysis with an anti-BrdU monoclonal antibody. Representative images of the hippocampal regions are shown in (A) and the quantification results of three BrdU^+^ progenitor populations, outlined as the primary matrix (1ry), secondary matrix (2ry) and tertiary matrix (3ry), are presented in (B). The tertiary matrix corresponds to the developing dentate gyrus. For each time point, the quantification was based on 2 pairs of control and mutant brains, with 4 or 5 matched sections per brain. (C-D) After injection with BrdU at P12, wild-type and mutant pups were sacrificed 1 h later for immunohistochemical analysis with the anti-BrdU antibody. Representative images of the hippocampal regions are shown in (C) and the quantification result of BrdU^+^ S-phase cells at the subgranular zone (marked with the yellow dashed lines) is presented in (D). The quantification was based on 2 pairs of control and mutant pups, with 4 matched sections per pup. Scale bars, 200 μm; ns, not statistically significant; **p<0.01; ***p<0.001.

At the prenatal and postnatal stages, dentate gyrus development involves two waves of neuronal precursors migrating from the neuroepithelium at the ventricular or subventricular zone to the dentate gyrus [58,59,74,75], so we next investigated whether and how Brpf1 loss affects the migration. For this, BrdU was injected into pregnant dams at E12.5, E14.5 and E16.5. The dams were then sacrificed at P0 to isolate the brain for immunohistochemical analysis with an anti-BrdU monoclonal antibody. BrdU^+^ cells were quantified as three populations, outlined as the primary matrix (1ry), secondary matrix (2ry) and tertiary matrix (3ry) (Fig. 7A, top left) [66]. Representative images of the hippocampal regions are shown in Fig. 7A and the quantification results of the three BrdU^+^ populations are presented in Fig. 7B. In the first and second matrices, no difference was found between the control and mutant for all three labeling time points. In the tertiary matrix (corresponding to the developing dentate gyrus), there were fewer BrdU^+^ cells when labeled at E12.5 and E14.5 (Fig. 7A-B), indicating that Brpf1 loss affects migration of neuronal progenitors from the dentate neuroepithelium to the developing dentate gyrus.

**Fig 8:**
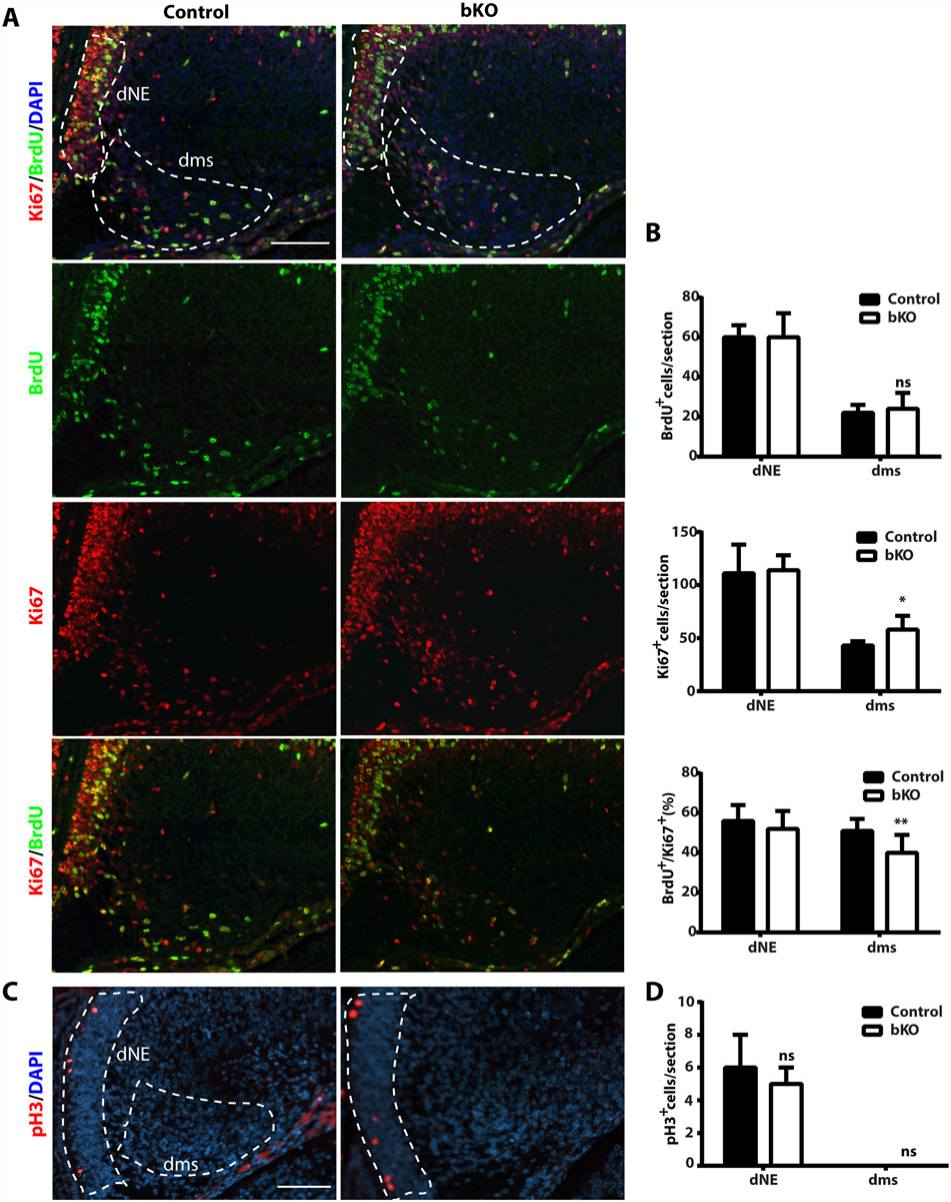
Cell cycle properties and progenitor number in the mutant dentate gyrus. (A-B) After BrdU labeling, E15.5 pregnant mice were sacrificed 1 h later to retrieve fetal brains for subsequent fixing and sectioning. Immunofluorescence microscopy was performed with anti-Ki67 and-BrdU antibodies, with representative images of the hippocampal regions shown in (A) and the quantification of the stained cells in two regions (outlined with dotted lines) presented in (B). The quantification was based on two pairs of control and mutant brains, with 8 matched sections per brain. In the dentate neuroepithelium (dNE), no difference was detected. In the dentate migration stream (dms), the number of BrdU^+^ progenitors was normal but the Ki67^+^ cycling cells increased significantly, thereby decreasing the ratio of S-phase (BrdU^+^) vs proliferating (Ki67^+^) cells. (C-D) Immunostaining of sections from the same brains as in (A-B) with an antibody specific to phospho-Ser10 of histone H3 (pH3). Representative images of the hippocampal regions are shown in (C) and the quantification of positive cells in two regions (outlined with dashed lines) is presented in (D). No pH3-positive cells were detected in the migration stream. Scale bars: 100 μm; ns, not statistically significant; *p<0.05, **p<0.01.

At both P10 and P24, Ki67^+^ neuronal precursors virtually disappeared in the subgranular zone of the mutant dentate gyri (Fig. 3D-E). To substantiate this, we performed BrdU labeling and sacrificed the pups 1 h later. Different from the birthdating analysis just described above (Fig. 7A-B), this short labeling protocol was to assess cells with active DNA synthesis. As shown in Fig. 7C-D, this protocol identified dramatic reduction of BrdU^+^ cells at the mutant subgranular zone at P12. In addition, immunostaining for cleaved caspase 3 failed to evident apoptosis in the wild-type or mutant dentate gyrus. Together, these results indicate that Brpf1 impairs cell cycle progression. To investigate this further, we analyzed cell cycle progression of the progenitors. E15.5 pregnant mice were sacrificed after 1 h pulse of BrdU labeling to retrieve the fetal brain for immunofluorescence microscopy with anti-Ki67 and-BrdU antibodies. Representative images of the hippocampal regions are shown in Fig. 8A and the quantification of the immunostained cells in two regions (outlined with dotted lines) is presented in Fig. 8B. In the dentate neuroepithelium, no difference was detected (Fig. 8B, left). In the dentate migration stream, the number of BrdU^+^ progenitors was normal but the Ki67^+^ cycling cell population (at G1, S, G2 and M, but not G0) increased significantly, thereby decreasing the ratio of S-phase (BrdU^+^) over proliferating (Ki67^+^) cells (Fig. 7B, right). Moreover, immunostaining analysis of the related sections with an antibody specific to phospho-Ser10 of histone H3 revealed no difference between the wild-type and mutant (Fig. 8C-D), indicating that the M phase of the cell cycle is normal in the mutant. These results indicate that Brpf1 loss impairs cell cycle progression of the dentate migration stream at E15.5, most likely through the G1 phase. Notably, the defects at E15.5 (Fig. 8) were smaller than those at or after P10 (Figs. 3D-E & 7C-D). Consistent with difference, Brpf1 expression in the developing hippocampus was low at E14.5 and E17.5, but increased dramatically after P3 (Fig. 1).

### Brpf1 loss deregulates transcription important for dentate gyrus development

**Fig 9:**
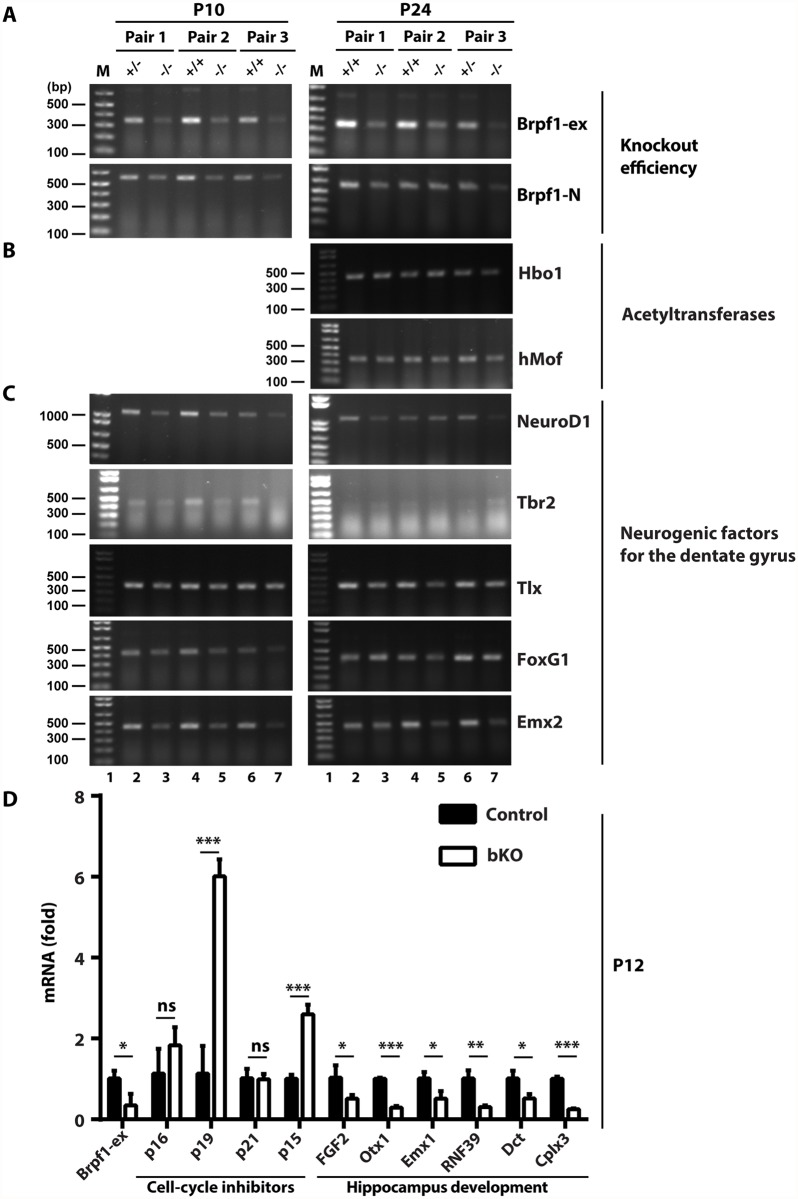
Brpf1 loss deregulates gene expression important for hippocampus development. (A-C) RT-PCR analysis of transcripts for Brpf1 (A), the acetyltransfrases Hbo1 and hMof (B), and five transcription factors (C), loss of which is known to cause dentate gyrus hypoplasia (see the text). The primers used are listed in S1 Table. Gapdh was used as the internal control [57]. At P24, Tbr2 expression is known to be limited to the subgranular zone of the dentate gyrus, so the RT-PCR product was not detected. (D) RT-qPCR analysis of transcripts for Brpf1, four cell cycle inhibitors and six genes important for hippocampus development. The p16/19, p15 and six other genes were identified in the microarray analysis of dorsal brain cortices isolated from three pairs of wild-type and mutant pups at P4 [57]. The RT-qPCR analysis was performed on three pairs of dorsal brain cortices at P12 and the average values are shown with standard deviation. Dct, dopachrome tautomerase; Cplx3, presynaptic protein complexin 3. *, *p*<0.05; **, *p*<0.01; ***, *p*<0.001; ns, not statistically significant.

Having identified the cellular mechanisms for the observed defects in the dentate gyrus, we then investigated the underlying molecular mechanisms. Brpf1 activates Moz, Morf and Hbo1 [30–32]. These acetyltransferases function as transcriptional coregulators [19,76–79]. Thus, we considered whether *Brpf1* inactivation deregulates transcription. For this, we first performed RT-PCR. *Brpf1* deletion occurred efficiently (Fig. 9A) [57]. The inactivation did not affect mRNA levels of Brpf2, Brpf3, Moz and Morf (S1D Fig). Similarly, neither Hbo1 nor hMof was altered (Fig. 9B). Importantly, mRNA levels of NeuroD1, Tbr2 and FoxG1 were reduced in the mutant (Fig. 9C). This is consistent with the immunofluorescence microscopic results Figs. (6 & S4). The transcript levels of Emx2 and Tlx were also reduced (Fig. 9C). As these transcription factors are known to be important for dentate gyrus development, these results nicely explain hypoplasia of the bKO dentate gyrus.

As Brpf1 loss affects cell cycle progression (Figs. 3D-E, 7C-D & 8), we analyzed expression of four cell cycle inhibitors, p16, p19, p21 and p15. Among them, p16 and p21 are two known targets of Moz [19,80]. Microarray-based gene expression analysis identified the *Cdkn2a* (encoding both p16 and p19) and *Cdkn2b* (encoding p15) genes as two of the top 35 candidates whose transcription was upregulated in the mutant dorsal cortex at P4 [57]. As shown in Fig. 9D, RT-qPCR identified increase in transcripts of p19 and p15, but not p16 or p21. These results indicate that Brpf1 loss promotes p15 and p19 transcription, which may then contribute to deregulated cell cycle progression. The microarray analysis [57] also identified transcriptional reduction in six genes related to hippocampus development (Fig. 9D). RT-qPCR confirmed this (Fig. 9D), supporting that Brpf1 is required for gene expression important for hippocampus development.

### Contribution of Moz, Morf and Hbo1 to effects of *Brpf1* deletion

As a scaffold, Brpf1 bridges subunit interaction, stimulates enzymatic activity and restricts substrate specificity of Moz, Morf and Hbo1 acetyltransferase complexes [30–32,41], so we asked whether these acetyltransferases contribute to the defects observed in the Brpf1 bKO mice. Related to this, mice with residual *Morf* expression display defects in the neocortex but not the hippocampus [53], suggesting that either Morf is not the sole mediator or it is not involved at all. Using a knockin *LacZ* reporter, we have recently detected Moz expression in the hippocampus, with a pattern similar to that of Brpf1 [55]. To investigate the role of Moz in the hippocampus and neocortex, we crossed *Moz* ^*f/+*^ mice with the *Emx1-Cre* strain to obtain *Moz* ^*f/+*^;*Emx1-Cre* mice. Intercrosses yielded *Moz* ^*f/f*^;*Emx1-Cre* mice. They appeared normal and Nissl staining of the brain sections revealed none of the defects observed in the *Brpf1*-deficient brain, suggesting that like Morf, Moz is either not the sole mediator of Brpf1 or not involved at all.

We then asked whether Hbo1 is involved. For this, we determined its expression in the hippocampus by immunostaining. At P10, Hbo1 was enriched in pyramidal layers of CA1 and CA3, as well as in the granular cell layer of the dentate gyrus (S5A Fig, left two panels). A similar expression pattern was also detected at P4 (S5B Fig, left two panels). When *Brpf1* was deleted, the expression of Hbo1 in these three regions became weak at P4 and almost disappeared at P10 (S5A-B Fig, right two panels). The difference was not obvious at E16.5 (S5C Fig). At P12, the total Hbo1 protein level (S5D Fig) at the dorsal cortex was not affected, suggesting that *Brpf1* deletion may destabilize Hbo1 specifically at the pyramidal layers of the CA regions and the granular cell layer of the dentate gyrus.

The MYST family of mammalian acetyltransferases is composed of five members: MOZ, MORF, HBO1, TIP60 and hMOF in mammals [16]. MOZ and MORF are almost interchangeable in cell-based assays [31], so we compared the interactions of MOZ, HBO1, TIP60 and hMOF with BRPF1 under the same experimental conditions. As shown in S6 Fig (lanes 1–2 & 5–6), co-expression of BRPF1, ING5 and EAF6, which are known to form a trimeric complex [31], increased the expression levels of MOZ and HBO1, possibly through tetrameric complex formation and subsequent stabilization. This may explain the loss of Hbo1 expression by immunofluorescence microscopy (S5A-B Fig). HBO1 was slightly more efficient than MOZ in co-precipitation of BRPF1 (S6 Fig). Interestingly, HBO1 co-precipitated both ING5 and EAF6 much more efficiently than MOZ, indicating that HBO1 forms a better tetrameric complex than MOZ. By contrast, TIP60 was much less efficient and hMOF displayed no affinity for BRPF1, ING5 and EAF6 (S6 Fig). These cell-based results indicate that although both MOZ and HBO1 interact efficiently and specifically with BRPF1, HBO1 is better than MOZ in forming a tetrameric complex with BRPF1, ING5 and EAF6.

## Discussion

Herein we have demonstrated that Brpf1 is dynamically expressed during forebrain development (Fig. 1) and that forebrain-specific inactivation of mouse *Brpf1* led to abnormalities in the hippocampus, esp. the dentate gyrus (Fig. 2), highlighting its importance in hippocampal neurogenesis. This is the first report of an epigenetic regulator whose loss exerts such profound effects on hippocampus development. Loss of two other groups of proteins has been reported to yield similar phenotypes. The first group includes eight DNA-binding transcription factors, Sox2 [61], Tlx (tailless) [62], Tbr2 [63], NeuroD1 [64], Emx2 [65], neurogenin 2 [66], Prox1 [81] and FoxG1 (also known as BF1, brain factor 1) [67]. Within the second group, there are three signaling regulators, Cxcr4, smoothened and Kif3a [70,82]. The latter two are important for Hedgehog signaling, while Cxcr4 is a receptor for the chemokine Cxcl12 (also known as stromal cell-derived factor 1). The Cxcl12-Cxcr4 signaling pair is not only important for granular neuron migration during dentate gyrus development [70], but also for the homing and self-renewal of hematopoietic stem cells [83]. Our unexpected finding that Brpf1 plays a similar role in the dentate gyrus suggests a potential link of the DNA-binding transcription factors and the signaling molecules to epigenetic and acetylation regulation by Brpf1. The similar phenotypes suggest that these two groups of regulators and Brpf1 define novel pathways required for patterning the dentate gyrus. The dentate gyrus develops from the cortical hem around mid-gestation, involving subsequent neuron migration and specification until the second week after birth [58,59,74,75]. Brpf1 appeared to regulate developmental processes at the late gestation and postnatal stages (Figs. 4–7). The dentate gyrus is also key to learning and memory, so it will be important to map out how each regulator is involved. The outcome will shed light on neurogenesis in the subgranular zone, a key area with adult neural stem cells [68,73].

The bKO mice are particularly similar to those lacking Tbr2 in terms of dentate gyrus hypoplasia [63]. Specific to intermediate neuronal progenitors, Tbr2 is required for transition from neural stem cells to these progenitors [72]. Since Tbr2^+^ cells were compromised by *Brpf1* inactivation (Fig. 6A-E), Brpf1 may be essential for progression from neural stem cells to the progenitors. Mouse Cxcl12 and its receptor Cxcr4 are important for migration of granular cells to the dentate gyrus [84,85] and Tbr2 controls Cxcr4 expression and regulates Cxcl12 signaling [70], so compromised Cxcl12-Cxcr4 signaling may also contribute to dentate gyrus hypoplasia. In addition, Hedgehog signaling may be involved. First, *Smoothened* loss causes dentate gyrus hypoplasia [82]. Second, the neural stem cell marker Sox2 is required for Hedgehog signaling and its loss results in similar hypoplasia [61]. Finally, *Brpf1* inactivation reduced Sox2 expression (Fig. 4A-D). Thus, Brpf1 is important for production of Sox2^+^ neural stem cells and Tbr2^+^ intermediate neuronal progenitors.

As for additional cellular mechanisms, Brpf1 loss led to abnormal migration of neuronal progenitors from the dentate neuroepithelium to the dentate gyrus (Fig. 7A-B). The cell cycle progression was also impaired (Figs. 3D-E, 7C-D & 8). At the molecular level, Brpf1 loss reduced transcription of NeuroD1, Tbr2, Tlx, FoxG1 and Emx2 (Fig. 9C). In addition, transcription of the cell cycle inhibitors p15 and p19 was elevated, whereas the transcript levels of six genes important for hippocampal development were reduced in the mutant (Fig. 9D). Further studies are needed to investigate how Brpf1 loss confers histone acetylation and chromatin changes at these specific loci and across the entire genome.

Brpf1 interacts with Moz, Morf and Hbo1, and stimulates their acetyltransferase activities [30–32], so an interesting question is how these acetyltransferases may mediate the influence of Brpf1, regulate transcription of genes such as Tbr2 and Sox2, and contribute to hippocampus development. Morf is expressed in the hippocampus, but its inactivation does not affect the hippocampus [53]. In the hippocampus, Moz and Hbo1 display expression patterns similar to Brpf1 (Figs. 1 & S5) [55]. All three acetyltransferases interact efficiently with Brpf1 [30–32], although Hbo1 is better than Moz and perhaps also Morf (a Moz paralog) in forming a tetrameric complex with Brpf1, Ing5 and Eaf6 (S6 Fig). Inactivation of either Moz or Morf does not exert the same effects on the hippocampus as *Brpf1* deletion [53]. Interestingly, immunofluorescence microscopy identified reduction of Hbo1 expression in the granular cell layer of the dentate gyrus (S5A-B Fig) even though the total protein level in the dorsal cortex was not altered (S5D Fig). The H3K14 acetylation level was not altered in the mutant dorsal cortex (S5D Fig). Related to this, deletion of the *Hbo1* gene in mouse embryos or its fibroblasts dramatically reduces the H3K14 acetylation level [41,79]. Thus, even if it has a role, Hbo1 may not be the sole mediator. Instead, Moz, Morf and Hbo1 may have redundant roles in the hippocampus, so Brpf1 may act through all of them. As human HBO1 was slightly better in forming a tetrameric complex with BRPF1, ING5 and EAF6 (S6 Fig), mouse Hbo1 may be a major contributor in mediating Brpf1 functions. Alternatively, Brpf1 may function independently of Moz, Morf and Hbo1. Related to this, in addition to a small region required for interaction with the three acetyltransferases, BRPF1 possesses two PHD fingers for binding to unmodified histone H3 [32], one bromodomain for acetyllysine-recognition [33] and a PWWP domain for interaction with methylated histone H3 [34,35]. It also possesses a motif for interaction with ING5 and EAF6 to form a trimeric complex [31], and ING5 has its own histone binding ability [86]. Global inactivation of *Hbo1* is embryonically lethal [79], but specific deletion in different tissues has not been carried out. Further analysis of single and compound brain-specific knockouts of Moz, Morf and Hbo1 will shed light on how Brpf1 may, or may not, act through these acetyltransferases during hippocampus development.

In summary, we have taken a mouse genetic approach and demonstrated that Brpf1 is important for development of the hippocampus, especially the dentate gyrus, one of the two major areas with active adult neurogenesis. Mechanistically, Brpf1 is important for proper development of related neural stem cells and intermediate progenitors by regulating neuronal migration, cell cycle progression and transcription of related genes. Further studies are needed to investigate its molecular and genetic interaction with related transcription factors, histone acetyltransferases and other chromatin regulators, and to determine the genome-wide action during hippocampus development.

## Methods

### Maintenance of mouse colonies and use of mice

Mouse strains were maintained in a newly established animal facility at McGill University and all procedures involving the use of mice were performed according to guidelines and protocols approved by the McGill Animal Use Committee.

### Generation of knockout mice

Heterozygous *Brpf1* ^*l*^ mice have been described [55]. A promoterless *LacZ* cassette is between two FRT sites, while two loxP sites flank exons 4–6 of Brpf1 (S1A Fig). For genotyping, genomic PCR with primers Brpf1-F1 and-R1 generated a 227-bp band for wild-type, whereas genomic PCR with primers Brpf1-F1 and-mR1 produced a 162-bp fragment for the knock-in allele (S1A Fig). The *LacZ* cassette was deleted by breeding with PGK1-FLPo mice (Jackson Laboratory) to obtain the conditional allele *Brpf1* ^*f*^ (S1A Fig). Cross of *Brpf1* ^*f/+*^ mice with *Emx1-Cre* mice (Jackson Laboratory) resulted in the heterozygote *Brpf1* ^*f/+;*^ *Emx1-Cre* (or *Brpf1* ^*+/-*^, S1A Fig), and subsequent intercrosses yielded the homozygote *Brpf1* ^*f/f;*^ *Emx1-Cre* (or *Brpf1* ^-/-^, referred to as bKO). The lines were maintained on the C57BL/6J background. Heterozygous *Moz* ^*l/+*^ mice have been described [55] and *Moz* ^*f/+*^ mice were generated as described above. Cross of *Moz* ^*f/+*^ mice with Emx1-Cre mice (Jackson Laboratory) resulted in *Moz* ^*f/+;*^ *Emx1-Cre*, and subsequent intercrosses yielded *Moz* ^*f/f;*^ *Emx1-Cre*. *Moz* lines were maintained on a mixed C57BL/6J-CD1 genetic background. Other experimental procedures are presented in S1 Text.

## Supporting Information

S1 Fig Forebrain-specific inactivation of the *Brpf1* gene. (A) Generation of the *Brpf1* ^*f/f*^;*Emx1-Cre* (bKO) mice. Mice heterozygous for the *Brpf1* ^*l*^ allele were crossed with PGK1-FLPo mice to remove the promoterless LacZ cassette and obtain the conditional *Brpf1* ^*f*^ allele. Through *Emx1-Cre* mediated recombination, the loxP-flanked region spanning exons 4–6 was deleted to yield *Brpf1* ^-^ allele. Mice heterozygous for *Brpf1* ^-^ allele were intercrossed to obtain *Brpf1* ^-/-^ bKO mice. The genotyping primers are indicated with small arrowheads. (B) Specific *Emx1-Cre* mediated excision of Brpf1 in the forebrain but not the cerebellum. The forebrain (the caudal part) and cerebellum were dissected out from wild-type (+/+), heterozygous (+/-) and homozygous (-/-) mutant mice at P10 for genomic PCR. Primers Brpf1-F1,-mR1 and-R1 were used to detect the wild type (227 bp), *Brpf1* ^*f*^ (440 bp) and *Brpf1* (162 bp) alleles. Primers Brpf1-F1 and-Ex03 were employed to amplify the *Brpf1* ^-^ allele (460 bp), whereas the primers Cre01 and Cre02 were used for detection of the *Emx1-Cre* sequence. The asterisk denotes non-specific bands (middle panel). F, the forebrain (the caudal part, including the hippocampus); C, cerebellum; M, 100 bp DNA ladder. (C) RT-PCR analysis of *Brpf1* mRNA. The forebrain and cerebellum were dissected out as in (B). A 339-bp fragment spanning the floxed exons (Brpf1-ex) was amplified to determine the specificity and efficiency of *Emx1-Cre* mediated excision, whereas a 577-bp fragment encoding the N-terminal part of Brpf1 (Brpf1-N) was used to assess the efficiency of inactivation of the entire transcript by nonsense mRNA decay. Gapdh was used as an internal control. The asterisk denotes non-specific bands (left panel). (D) Effect of *Brpf1* inactivation on transcription of Brpf2, Brpf3, Moz and Morf. RT-PCR was performed on the caudal cortices extracted from wild-type (+/+) and homozygous (-/-) mice at P10 as in (C).
(PDF)

S2 Fig Comparison of wild-type and mutant hippocampi by Timm’s stain. (A, C, D) Representative images of control and mutant hippocampi at P8. Three medial-to-lateral sagittal brains sections were prepared for staining and representative images of the hippocampal regions are shown here. Note that the mossy fibers of the suprapyramidal bundles and dentate hilum were missing in the mutant (marked with yellow asterisks). (B, E) Same as the wild-type images shown in (A) and (D), respectively, with annotations of different structures according to published atlases [87–91], with the following abbreviations: CC, corpus callosum; CA1, cornu ammonis; CA3, cornu ammonis 3; DG, dentate gyrus; dh, dentate hilum; EC, entorhinal cortex; Fi, fimbria; LV, lateral ventricle; SB, subiculum; spb, suprapyramidal bundles; PS, postsubiculum. Scale bars, 0.5 mm.
(PDF)

S3 Fig Expression of neurogenin 2 in wild-type and mutant hippocampi. (A) Immunofluorescence microscopy to detect Ngn2^+^ progenitors on P0 brain sections. The layers were not as well separated in the mutant section as in the wild-type. (B) Same as (A) except that the analysis was performed for P24 brain sections. In addition to the missing infrapyramidal blade of the dentate gyrus, the pyramidal layer in the mutant was loosely packed (compare the regions in the CA1 field marked with green arrowheads). Scale bars, 200 μm.
(PDF)

S4 Fig Expression of Tuj1 and FoxG1 during development of wild-type and mutant brains. (A) Immunostaining to detect Tuj1^+^(top) or FoxG1^+^ (bottom) neurons at the E13.5 neuroepithelium (NE). (B) Double staining to detect Tuj1^+^ and FoxG1^+^ neurons in wild-type and mutant brain sections at P0. The boxed areas denote the hippocampal regions. (C) Immunostaining to detect FoxG1^+^ neurons in P24 sections. In addition to the missing infrapyramidal blade of the dentate gyrus, FoxG1^+^ neurons appeared in the mutant CA3 field and were not as tightly packed in the mutant CA1 field when compared to the wild-type. Abbreviations: CA1, cornu ammonis 1; CA3, cornu ammonis 3; ib, infrapyramidal blade; HP, hippocampus; NE, neuroepithelium; sb, suprapyramidal blade. Scale bars, 200 μm.
(PDF)

S5 Fig Hbo1 expression in wild-type and mutant brains. (A-C) Immunofluorescence microscopy to detect wild-type and mutant hippocampi. At P10, Hbo1 expression was enriched in the wild-type pyramidal layers of CA1 and CA3, as well as in the granular layer of the dentate gyrus (A, left). This pattern was not present in the mutant (A, right). At P4, the difference between the wild-type and mutant sections was smaller (B). At E16.5, the difference between the wild-type and mutant was not evident (C). Green arrowheads mark strong staining at the ventricular zones and pink arrowheads denote either skin or a folded region. Scale bars, 200 μm. (D) Immunoblotting of protein extracts from three pairs of the P12 heterozygous and homozygous dorsal cortices (including the hippocampus) with the indicated antibodies. On the top immunoblot, the middle band corresponds to the expected size of Hbo1 while the other two bands may be isoforms specific to the brain.
(PDF)

S6 Fig Human BRPF1 preferentially interacts with MOZ and HBO1. Four members of the MYST family of human histone acetyltransferases (MOZ, HBO1, TIP60 and hMOF) were transiently expressed in HEK293 cells as Flag-tagged fusion proteins with or without the expression of HA-tagged BRPF1,-ING5 and-EAF6 as indicated. Protein extracts were prepared for affinity-purification on M2 agarose conjugated with the anti-Flag antibody (Sigma). After extensive washing, bound proteins were eluted with the Flag peptide for immunoblotting with the anti-Flag and-HA antibodies as specified. Note that expression of BRPF1 stabilized MOZ and HBO1 (compare lanes 1–2 with 5–6 on the top blot), and that BRPF1 expression increased levels of ING5 and EAF6 (compare lanes 10–11, bottom panel).
(PDF)

S1 Table List of RT-PCR and RT-qPCR primers. (PDF)

S1 Text Supplemental experimental procedures. (PDF)
