# Correction: The Lysine Acetyltransferase Activator Brpf1 Governs Dentate Gyrus Development through Neural Stem Cells and Progenitors

**DOI:** 10.1371/journal.pgen.1005329

**Published:** 2015-06-15

**Authors:** Linya You, Kezhi Yan, Jinfeng Zou, Hong Zhao, Nicholas R. Bertos, Morag Park, Edwin Wang, Xiang-Jiao Yang

The third author's name is spelled incorrectly. The correct name is: Jinfeng Zou. The correct citation is: You L, Yan K, Zou J, Zhao H, Bertos NR, Park M, et al. (2015) The Lysine Acetyltransferase Activator Brpf1 Governs Dentate Gyrus Development through Neural Stem Cells and Progenitors. PLoS Genet 11(3): e1005034. doi:10.1371/journal.pgen.1005034
