# Supplementary figures and images for: The Lysine Acetyltransferase Activator Brpf1 Governs Dentate Gyrus Development through Neural Stem Cells and Progenitors
Source: PLoS Genet. 2015 Mar 10;11(3):e1005034. doi: 10.1371/journal.pgen.1005034 (PMC4355587; doi:10.1371/journal.pgen.1005034)

**A**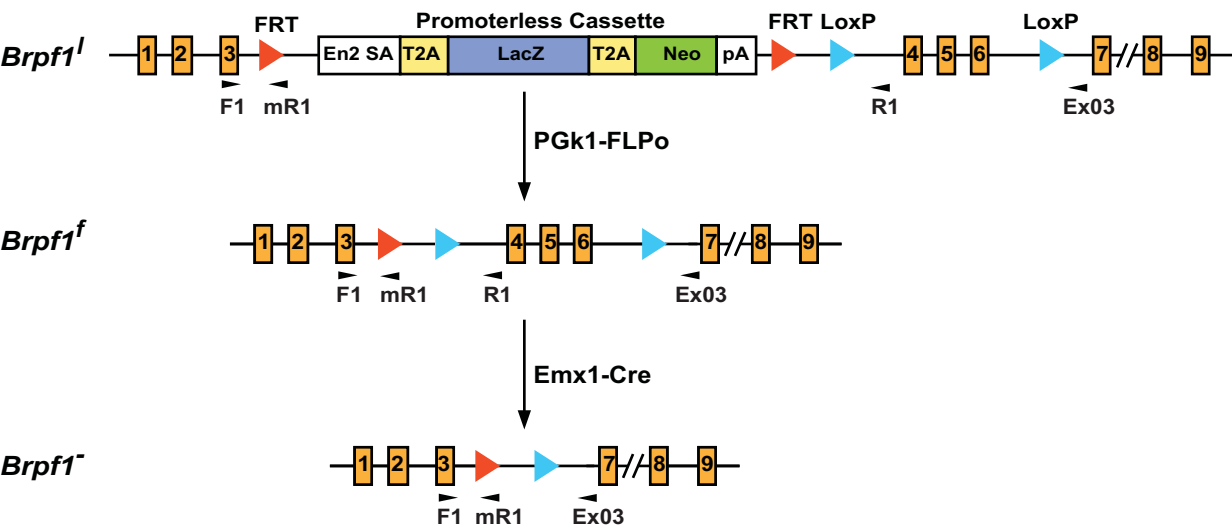**B**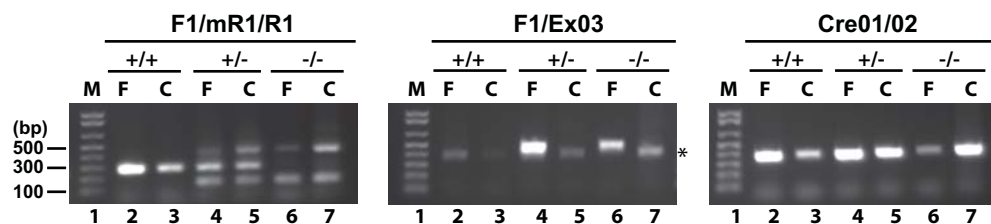**C**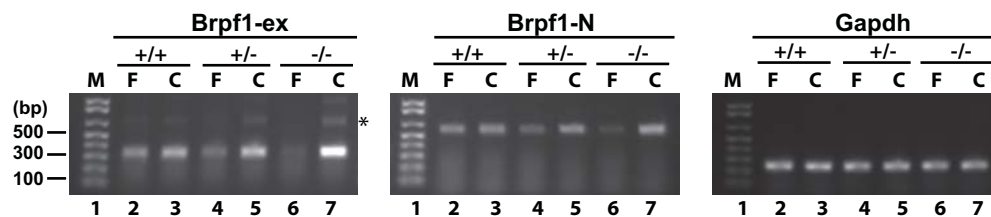**D**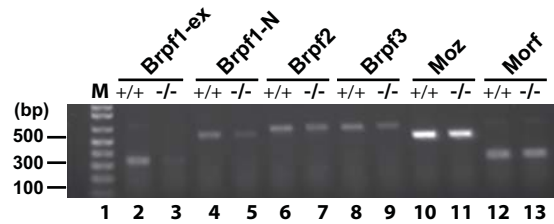

Supplement: S1 Fig — (A) Generation of the Brpf1 f/f;Emx1-Cre (bKO) mice. Mice heterozygous for the Brpf1 l allele were crossed with PGK1-FLPo mice to remove the promoterless LacZ cassette and obtain the conditional Brpf1 f allele. Through Emx1-Cre mediated recombination, the loxP-flanked region spanning exons 4–6 was deleted to yield Brpf1 - allele. Mice heterozygous for Brpf1 - allele were intercrossed to obtain Brpf1 -/- bKO mice. The genotyping primers are indicated with small arrowheads. (B) Specific Emx1-Cre mediated excision of Brpf1 in the forebrain but not the cerebellum. The forebrain (the caudal part) and cerebellum were dissected out from wild-type (+/+), heterozygous (+/-) and homozygous (-/-) mutant mice at P10 for genomic PCR. Primers Brpf1-F1,-mR1 and-R1 were used to detect the wild type (227 bp), Brpf1 f (440 bp) and Brpf1 (162 bp) alleles. Primers Brpf1-F1 and-Ex03 were employed to amplify the Brpf1 - allele (460 bp), whereas the primers Cre01 and Cre02 were used for detection of the Emx1-Cre sequence. The asterisk denotes non-specific bands (middle panel). F, the forebrain (the caudal part, including the hippocampus); C, cerebellum; M, 100 bp DNA ladder. (C) RT-PCR analysis of Brpf1 mRNA. The forebrain and cerebellum were dissected out as in (B). A 339-bp fragment spanning the floxed exons (Brpf1-ex) was amplified to determine the specificity and efficiency of Emx1-Cre mediated excision, whereas a 577-bp fragment encoding the N-terminal part of Brpf1 (Brpf1-N) was used to assess the efficiency of inactivation of the entire transcript by nonsense mRNA decay. Gapdh was used as an internal control. The asterisk denotes non-specific bands (left panel). (D) Effect of Brpf1 inactivation on transcription of Brpf2, Brpf3, Moz and Morf. RT-PCR was performed on the caudal cortices extracted from wild-type (+/+) and homozygous (-/-) mice at P10 as in (C). (PDF) [file pgen.1005034.s001.pdf]

Figure S2

A

Control

bKO

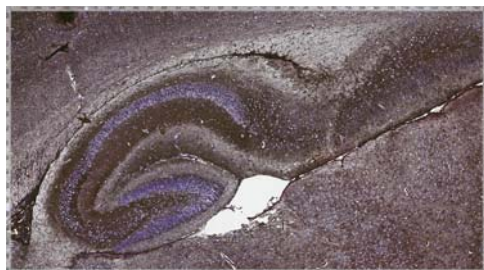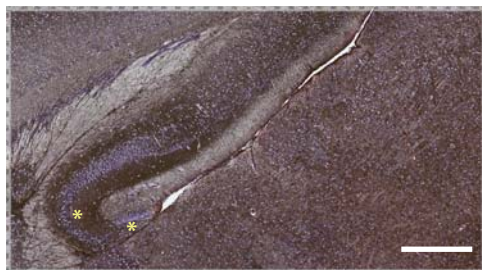

Medial

B

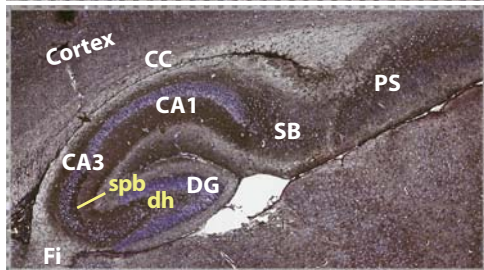

C

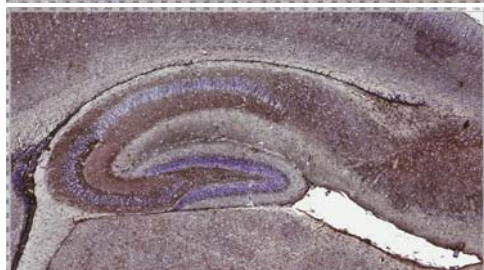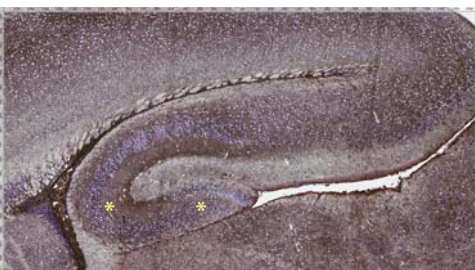

D

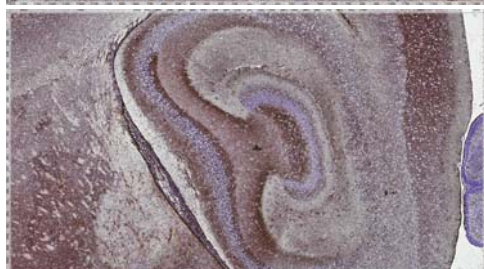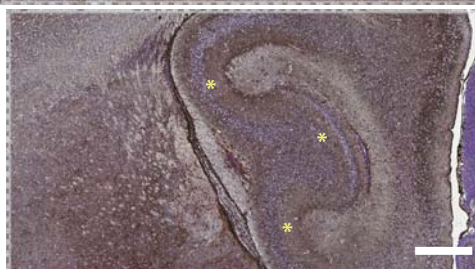

Lateral

E

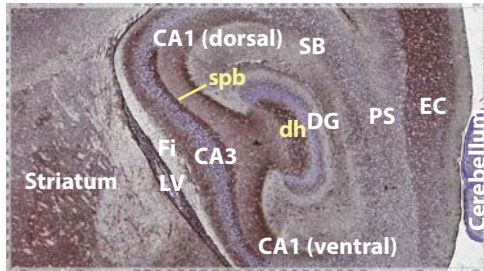

Supplement: S2 Fig — (A, C, D) Representative images of control and mutant hippocampi at P8. Three medial-to-lateral sagittal brains sections were prepared for staining and representative images of the hippocampal regions are shown here. Note that the mossy fibers of the suprapyramidal bundles and dentate hilum were missing in the mutant (marked with yellow asterisks). (B, E) Same as the wild-type images shown in (A) and (D), respectively, with annotations of different structures according to published atlases [87–91], with the following abbreviations: CC, corpus callosum; CA1, cornu ammonis; CA3, cornu ammonis 3; DG, dentate gyrus; dh, dentate hilum; EC, entorhinal cortex; Fi, fimbria; LV, lateral ventricle; SB, subiculum; spb, suprapyramidal bundles; PS, postsubiculum. Scale bars, 0.5 mm. (PDF) [file pgen.1005034.s002.pdf]

Figure S3

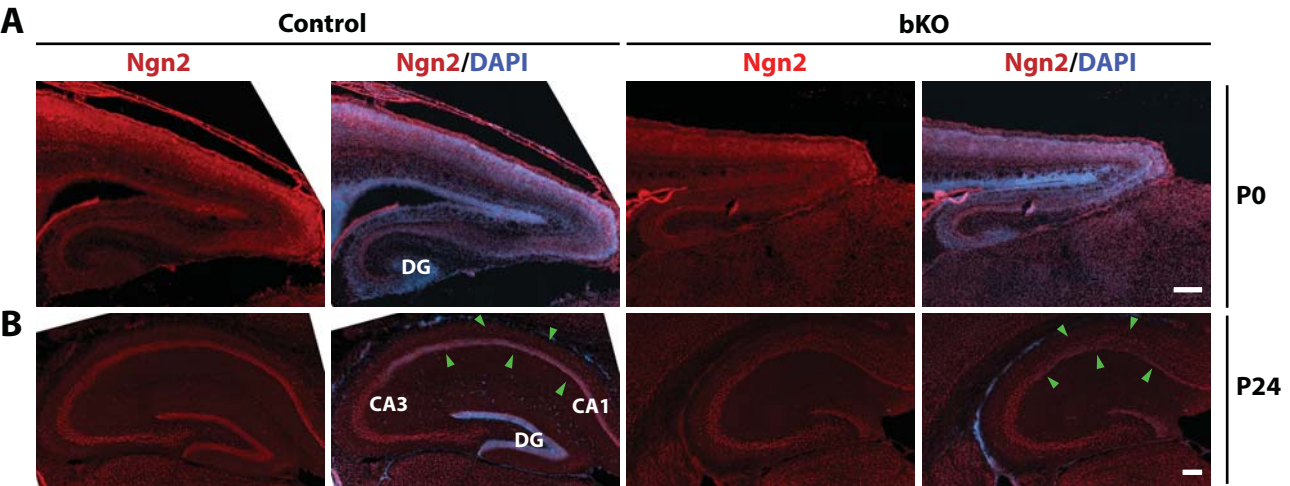

Supplement: S3 Fig — (A) Immunofluorescence microscopy to detect Ngn2+ progenitors on P0 brain sections. The layers were not as well separated in the mutant section as in the wild-type. (B) Same as (A) except that the analysis was performed for P24 brain sections. In addition to the missing infrapyramidal blade of the dentate gyrus, the pyramidal layer in the mutant was loosely packed (compare the regions in the CA1 field marked with green arrowheads). Scale bars, 200 μm. (PDF) [file pgen.1005034.s003.pdf]

Figure S4

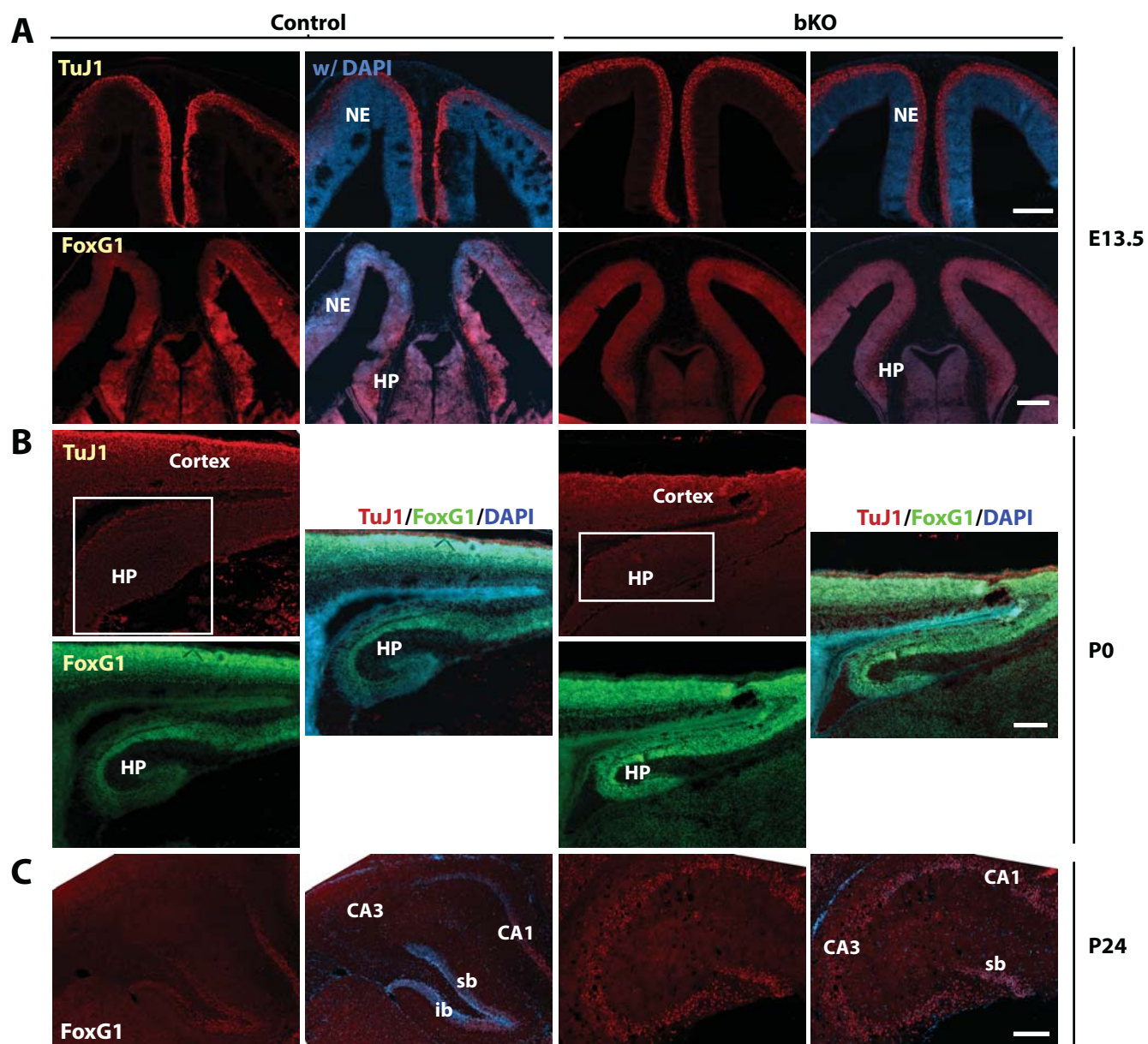

Supplement: S4 Fig — (A) Immunostaining to detect Tuj1+(top) or FoxG1+ (bottom) neurons at the E13.5 neuroepithelium (NE). (B) Double staining to detect Tuj1+ and FoxG1+ neurons in wild-type and mutant brain sections at P0. The boxed areas denote the hippocampal regions. (C) Immunostaining to detect FoxG1+ neurons in P24 sections. In addition to the missing infrapyramidal blade of the dentate gyrus, FoxG1+ neurons appeared in the mutant CA3 field and were not as tightly packed in the mutant CA1 field when compared to the wild-type. Abbreviations: CA1, cornu ammonis 1; CA3, cornu ammonis 3; ib, infrapyramidal blade; HP, hippocampus; NE, neuroepithelium; sb, suprapyramidal blade. Scale bars, 200 μm. (PDF) [file pgen.1005034.s004.pdf]

Figure S5

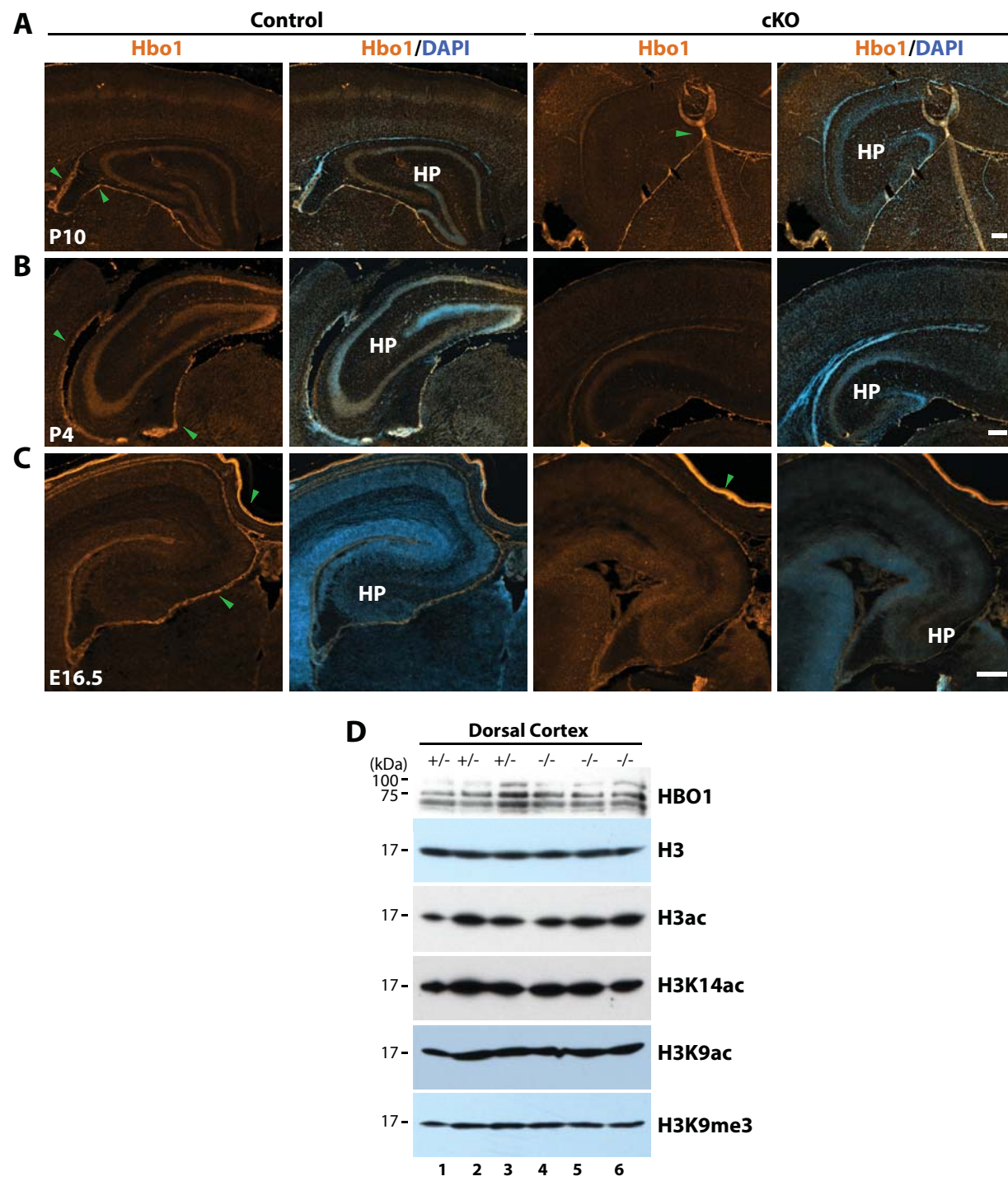

Supplement: S5 Fig — (A-C) Immunofluorescence microscopy to detect wild-type and mutant hippocampi. At P10, Hbo1 expression was enriched in the wild-type pyramidal layers of CA1 and CA3, as well as in the granular layer of the dentate gyrus (A, left). This pattern was not present in the mutant (A, right). At P4, the difference between the wild-type and mutant sections was smaller (B). At E16.5, the difference between the wild-type and mutant was not evident (C). Green arrowheads mark strong staining at the ventricular zones and pink arrowheads denote either skin or a folded region. Scale bars, 200 μm. (D) Immunoblotting of protein extracts from three pairs of the P12 heterozygous and homozygous dorsal cortices (including the hippocampus) with the indicated antibodies. On the top immunoblot, the middle band corresponds to the expected size of Hbo1 while the other two bands may be isoforms specific to the brain. (PDF) [file pgen.1005034.s005.pdf]

# Figure S6

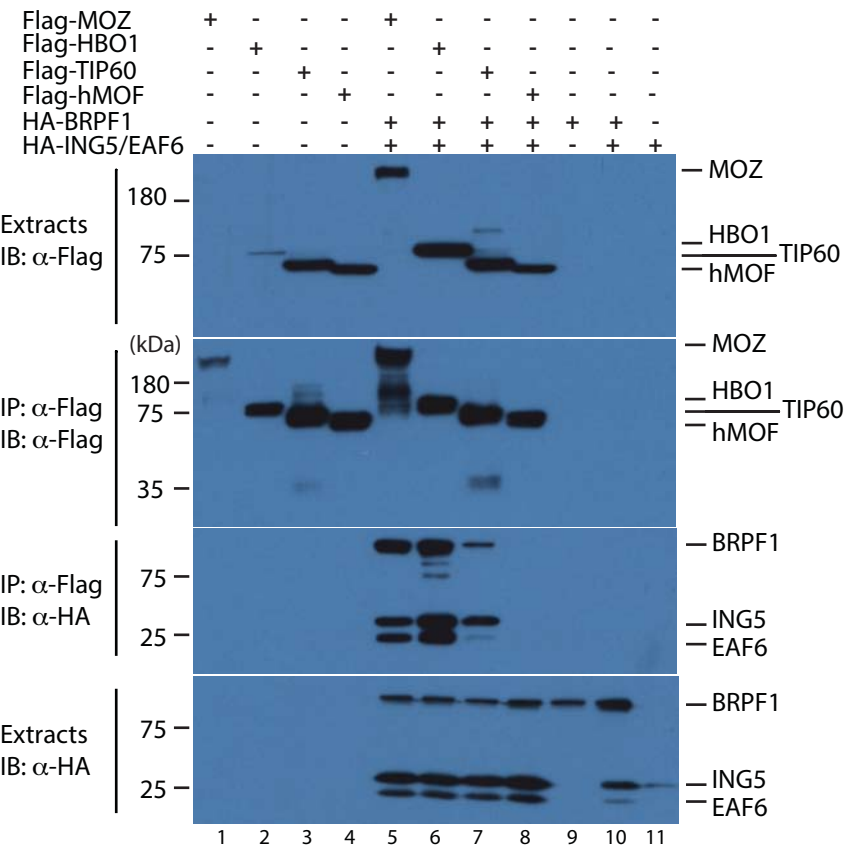

Supplement: S6 Fig — Four members of the MYST family of human histone acetyltransferases (MOZ, HBO1, TIP60 and hMOF) were transiently expressed in HEK293 cells as Flag-tagged fusion proteins with or without the expression of HA-tagged BRPF1,-ING5 and-EAF6 as indicated. Protein extracts were prepared for affinity-purification on M2 agarose conjugated with the anti-Flag antibody (Sigma). After extensive washing, bound proteins were eluted with the Flag peptide for immunoblotting with the anti-Flag and-HA antibodies as specified. Note that expression of BRPF1 stabilized MOZ and HBO1 (compare lanes 1–2 with 5–6 on the top blot), and that BRPF1 expression increased levels of ING5 and EAF6 (compare lanes 10–11, bottom panel). (PDF) [file pgen.1005034.s006.pdf]
