# Supplementary material for: The Lysine Acetyltransferase Activator Brpf1 Governs Dentate Gyrus Development through Neural Stem Cells and Progenitors
Source: PLoS Genet. 2015 Mar 10;11(3):e1005034. doi: 10.1371/journal.pgen.1005034 (PMC4355587; doi:10.1371/journal.pgen.1005034)
Supplement: S1 Table — (PDF) [file pgen.1005034.s007.pdf]

S1 Table List of RT-PCR and RT-qPCR primers

Primers for RT-PCR				
Name	Forward	Reverse	Length	ID
Brpf1-ex (floxed region)	CAGTAAGATCACCAACCGCC	GAGGAAAGGGGTCAGCTGCA	1711-2050	NM_030178.1
Brpf1-N (N-terminal)	CAGCCCCTCTGAAGTCTCAC	CTAGTGCATTGGGGTCACCT	379-956	NM_030178.1
Brpf2	CCAACCCTCAACCCAGTAGA	TATCAGCTCGCTTCGGTCTT	2742-3377	NM_001033274.3
Brpf3	CCACAGCTCCTGTCACTGAA	GTCACGCCTGTCTCTTCCTC	2621-3255	NM_001081315.1
Kat6a	ACCACCTACGAATGCTGGAC	ACTTTTCCTCGCAGTCTCCA	2686-3216	NM_001081149.1
Kat6b	TTGTTGTCTGTAACCAGTGA	GAATTCACACAGGTAAAGCT	1361-1687	NM_001205241.1
Hbo1	GCCGGCAATGCCGCGAAGGA	GGCTGGAGATGTCTATGTCA	191-630	NM_001195003
hMof	CCGGATAGCACCTGGCATTC	CATACTTCACTTTGGTGATC	211-550	NM_026370
Emx2	GCCACACCCCCTCTTCGCCT	TTTCCCAAGCTTTTAATCGTCTGAGGTCAC	850-1280	NM_010132
Tbr2	CGGCACCAAACTGAGATGAT	TGTGTCTCTGAGAAGGTGAA	1330-1760	NM_010136
NeuroD1	TTTCCC GAATTCACCAAATCATACAGCGAGAG	TTTCCCGGATCCTCTAATCGTGAAAGATGG	101-1174	NM_010894.2
FoxG1	GAGGCCGTCCAGAACGACAA	TCTCCTTCTCGTCCGGCCCG	641-980	NM_008241.2
Tlx	GTGTCTGCCACTCCTGAACG	TCGGAGCTGTCTGAACCGAG	1120-1520	NM_152229
p21	CACAGCGACCATGTCCAATC	GCGGGGCTCCCGTGGGCACT	201-680	U09507.1
Gapdh	TGATGACATCAAGAAGGTGGTGAA	TCTTACTCCTTGGAGGCCATGT	814-1059	XM_001476707.

				3
Primers for RT-qPCR				
Name	Forward	Reverse	Length	ID
Brpf1-ex	CAGTAAGATCACCAACC GCC	GAGGAAAGGGGTCAGCTGC A	1711-2050	NM_030178.1
p16	GGGTTTCGCCCAACGCC CCGA	TGCAGCACCACCAGCGTGT CC	190	NM_00104065 4
p19	GTTTTCTTGGTGAAGTTC GTGC	TCATCACCTGGTCCAGGATT C	134	NM_009877
p15	CCCTGCCACCCTTACCA GA	CAGATACCTCGCAATGTCA CG	204-372	NM_007670
p21	CACAGCGACCATGTCCA ATC	GCGGGGCTCCCGTGGGCAC T	201-680	U09507.1
FGF2	GCGACCCACACGTCAAA CTA	TCCCTTGATAGACACAACTC CTC	164-225	NM_008006
Otx1	ATGTCTTACCTCAAACA ACCCCC	GTAGCGAGTCTTTGCGAAC AG	4-186	NM_011023
Emx1	GAAGAATCACTACGTGG TGGG	CCGTTTGTATTTTGTCCTCC GA	537-648	NM_010131
RNF39	GTGGGCTGCGTGAGAAA CT	GTGCTGGGACATCGAATCG T	266-403	NM_001099632
Dct	TTCTGCTGGGTTGTCTGG G	CACAGATGTTGGTTGCCTCG	23-157	NM_010024
Cplx3	AAGGGGGACGGAGACA AGT	CTGTGCATCTCGCTCCATCT T	76-183	NM_146223
