# Supplementary material for: The Lysine Acetyltransferase Activator Brpf1 Governs Dentate Gyrus Development through Neural Stem Cells and Progenitors
Source: PLoS Genet. 2015 Mar 10;11(3):e1005034. doi: 10.1371/journal.pgen.1005034 (PMC4355587; doi:10.1371/journal.pgen.1005034)
Supplement: S1 Text — (PDF) [file pgen.1005034.s008.pdf]

S1 Text Supplemental experimental procedures

Tissue preparation

Animals were anesthetized with isoflurane inhalation before tissue collection. Pups and adults were perfused transcardially with cold PBS, followed by 4 % paraformaldehyde (PFA) in PBS. Pregnant dams were anesthetized and fetuses were sacrificed by decapitation. Brains were immersion-fixed in 4% PFA in PBS for 2-3 h at 4°C and cryoprotected in 30% sucrose before cryosectioning. Cryosections were prepared with a cryotome (Thermo Electron, 77200187) at appropriate thickness and air-dried before storage at -80°C. Alternatively, brains were fixed in 4% PFA for 24-48 h at 4°C, dehydrated and then embedded in paraffin. Paraffin sections were prepared on a microtome (Leica, RM2235) at 5 µm thickness and baked at 37°C overnight for storage at room temperature.

X-gal staining

The procedure was performed as described [55], with some modifications. Briefly, embryos or fetal brains were fixed in 1% PFA at 4ºC for 1 h. Postnatal brains were perfused with cold PBS and then with 2% cold PFA in PBS, and were subsequently post-fixed in 4% PFA at 4 ºC for 2 h. For X-gal staining of sections, tissues were cryoprotected in PBS/30% sucrose at 4ºC overnight until the samples sank to the bottom, and then embedded in Tissue-Tek OCT compound (Sakura Finetek, 4583) on dry ice for storage at -80ºC. 15 µm sections were prepared on a cryotome (Thermo Electron, 77200187).

Nissl staining

Nissl staining was performed as described [92]. Briefly, cryosections were prepared as above and air-dried before staining. Paraffin sections were dewaxed by incubation in xylene twice, 5 min each, and then rehydrated in a gradient of ethanol. Cryosections or dewaxed paraffin sections were stained in 0.1% cresyl violet solution (with 0.3 ml glacial acetic acid in 100ml solution and filter) for 6-8 min, rinsed in dH_2O, dehydrated in a gradient of ethanol (70%, 85%, 95% and 100%) for 3 min each, cleared in xylene and mounted with coverslips and ClearMount (American MasterTech Scientific, MMC0126). Slides were digitized with a Scanscope (Aperio, ScanScope XT) for further analysis.

Timm's stain

Timm's method for staining for zinc ions was performed according to an online protocol (Nikki Sunnen, BCM 4/09). Briefly, wild-type and mutant P8 pups were anesthetized with isoflurane and perfused transcardially with cold PBS, followed by a buffered sulfide solution containing 1.2% Na_2S. The brains were removed and immersed in the same perfusate for 1 h. After rinsing with dH_2O, the brains were fixed in 10% neutrally-buffered formalin (Sigma, HT501128) for 24 h and post-fixed in 3% glutaraldehyde in 30% dextrose solution for 1.5 h. Samples were embedded in paraffin for preparation of 5 μm serial sagittal sections. Dewaxed and rehydrated sections were stained in a preheated (26ºC) Timm's stain solution (30% gum arabic, 1.7% hydroquinone and 0.085% silver nitrate in citrate buffer) for 45 min in dark at room temperature and then for 20 min or more at 60ºC. Slides were counterstained in 0.1% cresyl violet as above and mounted with coverslips for examination under a light microscope.

Golgi-Cox staining

Wild-type and mutant P19 mice were anesthetized with isoflurane and perfused transcardially with cold PBS. Golgi-Cox impregnation was performed with the FD Rapid Golgi-Stain kit (FD NeuroTechnologies, PK401A) according to the manufacturer's instructions. 180 μm coronal slices were prepared on a vibratome (BioRad/EM Corp., Micro-Cut H1200). Black-white photographs under a bright field were taken with a microscope (Zeiss, Axio Observer Z1) controlled by the Zen software package (Zeiss).

Neuronal migration assays by BrdU labeling

Pregnant mice were injected intraperitoneally with 50 μg BrdU/g body weight at E12.5, E14.5 and E16.5. Brains from newborns were collected at P0, fixed in 4% PFA for 24 h and embedded in paraffin. 5 μm sections were prepared on a microtome. After dewaxing and rehydrating, sections were antigen-retrieved by boiling in 10 mM of sodium citrate at pH6.0 for 20 min before exposure to 2N HCl at 37°C for 30 min. Sections were then blocked with 2% BSA and 0.2% Triton X-100 in PBS (blocking buffer) at room temperature for 1 h before incubation with a rat anti-BrdU antibody (Abcam, AB6326, 1:100) in the blocking buffer at 4°C overnight. Sections were subsequently washed in PBS, and incubated in biotin-SP-conjugated AffiniPure donkey anti-rat IgG(H+L) (Jackson ImmunoResearch, 712-065-153, 1:200) in the blocking buffer for 1 h at room temperature. The signal was detected by the Vector ABC kit (Vector Labs, PK-4001) and DAB substrate kit (Vector Labs, SK-4100). Stained sections were digitized on a Scanscope (Aperio, ScanScope XT) with a 20x objective. The migration matrix and

developing dentate gyrus were divided into three regions as illustrated in Fig. 7A for manual counting of $BrdU^+$ cells. Only darkly labeled nuclei were manually counted; during counting, the genotypes were intentionally ignored to avoid bias. Four or five matched sections were analyzed for each pair of wild-type and mutant newborns.

Cell cycle analysis by BrdU labeling

For S phase analysis at P12, pups were injected with 50 μg BrdU/g body weight and sacrificed 1 h later. The brains were processed for anti-BrdU immunohistochemistry as described as above. $BrdU^+$ cells were manually counted in the subgranular zone of the dentate gyrus. Four matched sections were analyzed for each pair of wild-type and mutant pups.

For cell cycle analysis at E15.5, pregnant mice were injected with 50 μg BrdU per g of body weight intraperitoneally and fetal brains were collected 1 h later. Paraffin sections were used for immunofluorescence microscopy with anti-Ki67 and -BrdU antibodies. After antigen retrieval by boiling in 10 mM of sodium citrate at pH6.0 for 20 min, the sections were blocked and incubated simultaneously with rat anti-BrdU antibody (Abcam, AB6326, 1:100) and mouse anti-Ki67 antibody (BD Pharmingen, 556003, 1:400) at 4°C overnight. Alexa Fluor 488-conjugated goat anti-rat IgG (Invitrogen, A11006, 1:500) and Alexa Fluor 568-conjugated goat anti-mouse IgG (Invitrogen, A11031, 1:500) were used as the secondary antibodies. DAPI was used to stain the nuclei. The dentate neuroepithelium and migration stream were outlined as in Fig. 8A for manual counting of $BrdU^+$ and/or $Ki67^+$ cells in the regions; during counting, the genotypes were intentionally ignored to avoid bias. Eight matched sections were analyzed for each pair of wild-type and mutant embryos.

Indirect immunofluorescence microscopy

Indirect immunofluorescence microscopy was performed as described [92], with some modifications. Briefly, paraffin sections were antigen retrieved by boiling in 10 mM of sodium citrate at pH6.0 for 20 min, blocked with the blocking solution (2% BSA and 0.2% Triton X-100 in PBS) for 1 h at room temperature, and incubated in the primary antibody (diluted in the blocking solution) overnight at 4°C. Sections were subsequently washed in PBS, and incubated in the secondary antibody in the blocking solution for 1-2 h at room temperature, and washed with PBS. The sections were counterstained with 1 ng/ml DAPI (Sigma-Aldrich, D9542) for 5 min when necessary, and finally mounted with coverslips and the Immu-Mount mounting medium (Thermo Electron, 9990402). After drying overnight in dark, the slides were sealed with nail polish and examined under a fluorescence microscope (Zeiss, Axio Observer Z1) controlled by the Zen software package (Zeiss). The following antibodies were used: rabbit anti-glial fibrillary acidic protein (Gfap) (Dako, Z0334, 1:2000), rabbit anti-Tbr2/Eomes (Abcam, ab23345, 1:400); goat anti-NeuroD1 (Santa Cruz Biotech, sc-1084, 1:50-1:200), Goat anti-Sox2 (R&D systems, AF2018, 1:200), goat anti-doublecortin (Dcx) (Santa Cruz Biotech, sc-8066, 1:200), mouse anti-neuronal class III β-tubulin (Tuj1) (Covance, MMS-435P, 1:1000), rabbit anti-FoxG1 (Abcam, ab151556, 1:400) [93], goat anti-Hbo1 (Santa Cruz Biotech., sc-13284, 1:50), rat anti-Ctip2 (Abcam, ab18465, 1:400), mouse anti-phospho Ser10 histone H3 (USBiological Life Sciences, H5110-13K, 1:100), rabbit anti-cleaved caspase 3 (Asp175) (Cell signaling, 9661, 1:200), Alexa Fluor 568-conjugated goat anti-rabbit IgG (Invitrogen, A11011, 1:500), Alexa Fluor 488-linked goat anti-rabbit IgG

(Invitrogen, A11034, 1:500), Alexa Fluor 568-labeled goat anti-mouse IgG (Invitrogen, A11031, 1:500), Alexa Fluor 488-labeled goat anti-rat IgG (Invitrogen, A11006, 1:500) and Cy3-conjugated anti-goat IgG (Molecular Probes, 1:500) antibodies.

RT-PCR

RT-PCR was performed as described [55]. Briefly, total RNA was isolated from control and mutant caudal cortices using the Trizol reagent (Invitrogen, 15596018) and 1μg RNA was used for reverse transcription with the QuantiTect Reverse Transcription Kit (GIAGEN, 205311). Cycling conditions were as follows: 95ºC x 5 min, 28 cycles (95ºC x 15 sec, 50ºC x 15 sec and 72ºC x 45 sec) and 72ºC x 7 min. The reactions were carried out in a final volume of 10 μl containing the 2x GoTaq Green Master Mix (Promega, M7122) and 0.7 μM of each primer in RNase-free water. The relative levels between samples were normalized with the Gapdh level. Primers were designed, with aid of MacVector and the UCSC Genome Browser, and synthesized by IDT (USA). The primer sequences are listed in Table S1. Wherever possible, amplified regions with introns were used to avoid potential amplification artifacts from contaminated genomic DNA. In addition, regions with high GC content were avoided as much as possible.

RT-qPCR. Total RNA was isolated from 3 pairs of control and mutant caudal cortices at P12 with the miRNeasy Mini kit (QIAGEN). The RNA was reverse-transcribed by use of the Quantitect Reverse Transcription kit (QIAGEN). Real-time PCR was performed on Realplex2 (Eppendorf) with the Green-2-Go qPCR Mastermix (BioBasic). Primer sequences were taken from PrimerBank (http://pga.mgh.harvard.edu/primerbank/) or

designed on the web-based IDT RT-qPCR primer designer. Primers were synthesized by IDT Biotechnology and their sequences are listed in S1 Table.

Western blotting

For analysis of Hbo1 expression or histone acetylation by immunoblotting, 3 pairs of P12 control and bKO mice were anesthetized and decapitated. The brain caudal cortices were immediately dissected out, flash-frozen on dry ice and stored at -80°C. For homogenization, 500 μl of RIPA buffer (150 mM NaCl, 50 mM Tris-HCl pH8.0, 1% Triton X-100, 0.5% sodium deoxycholate, 0.1% SDS, 1 μg/ml pepstatin, 2 μg/ml aprotinin, 5 μg/ml leupeptin and 1 mM PMSF) was added rapidly to 0.1 g tissue on ice. The tissue was crushed thoroughly with a sterile blue pestle on ice and sonicated with a VirSonic 100 sonicator (Virtis Corp.) at the setting of 5 for 20 sec on ice. The sonicated suspension was then rotated at 4ºC for 1 h and centrifugated at 4°C and 16,000 rpm for 20 min. The supernatant was transferred to a fresh tube and was used immediately or divided into aliquots for flash-freezing and subsequent storage at -80°C. A small amount of the supernatant was used to determine the protein concentration with the BioRad protein assay reagent (Cat. 500-0006). For Western blotting, 40 μg of protein lysate was loaded per lane for separation by SDS-PAGE and transfer onto nitrocellulose membranes (Pall Corp., P/N66485). Membranes were blocked at room temperature for 1 h in the blocking solution [PBST (0.15% Tween 20 in PBS) containing 5% nonfat milk powder] and then incubated overnight at 4°C with rabbit anti-histone H3 (Abcam, ab1791, 1:5000), anti-acetyl-histone H3 (Lys14) (Millipore, 07-353, 1:2000), anti-histone H3 (acetyl K9) (Abcam, ab10812, 1:2000), anti-acetyl-histone H3 (Millipore, 06-599,

1:2000), anti-histone H3 (tri-methyl K9) (Abcam, 8898, 1:2000), or goat anti-HBO1 (Santa Cruz, sc-13284, 1:100) antibody diluted in the blocking solution. Blots were washed six times (5 min each) in PBST and incubated with horseradish peroxidase-conjugated donkey anti-rabbit secondary antibody (GE Healthcare, NA934V) or donkey anti-goat secondary antibody (Jackson ImmunoResearch, 705-035-147) diluted to 1:5000 in the blocking solution. Blots were further washed four times (5 min each) in PBST and developed with the enhanced chemiluminescence substrates (FroggaBio, 16024).

Immunoprecipitation

Immunoprecipitation was carried out as described [31]. Briefly, four members of the MYST family of human histone acetyltransferases (MOZ, HBO1, TIP60 and hMOF) were transiently expressed in HEK293 cells as Flag-tagged fusion proteins with or without the expression of HA-tagged BRPF1, -ING5 and -EAF6 as indicated. 48 h after transfection, protein extracts were prepared in buffer K for affinity-purification on M2 agarose conjugated with the anti-Flag antibody (Sigma). After extensive washing, bound proteins were eluted with the Flag peptide for immunoblotting with the anti-Flag and -HA antibodies as specified.

Supplemental References

92. Kim GW, Li L, Gorbani M, You L, Yang XJ (2013) Mice lacking alpha-tubulin acetyltransferase 1 are viable but display alpha-tubulin acetylation deficiency and dentate gyrus distortion. J Biol Chem 288: 20334-20350.
93. Marcal N, Patel H, Dong Z, Belanger-Jasmin S, Hoffman B, et al. (2005) Antagonistic effects of Grg6 and Groucho/TLE on the transcription repression activity of brain factor 1/FoxG1 and cortical neuron differentiation. Mol Cell Biol 25: 10916-10929.
